# Group A *Streptococcus* M1T1 Intracellular Infection of Primary Tonsil Epithelial Cells Dampens Levels of Secreted IL-8 Through the Action of SpyCEP

**DOI:** 10.3389/fcimb.2018.00160

**Published:** 2018-05-17

**Authors:** Amelia T. Soderholm, Timothy C. Barnett, Othmar Korn, Tania Rivera-Hernandez, Lisa M. Seymour, Benjamin L. Schulz, Victor Nizet, Christine A. Wells, Matthew J. Sweet, Mark J. Walker

**Affiliations:** ^1^School of Chemistry and Molecular Biosciences, University of Queensland, Brisbane, QLD, Australia; ^2^Wesfarmers Centre for Vaccines and Infectious Diseases, Telethon Kids Institute, University of Western Australia, Perth, WA, Australia; ^3^Australian Institute for Bioengineering and Nanotechnology, University of Queensland, Brisbane, QLD, Australia; ^4^Australian Infectious Diseases Research Centre, University of Queensland, Brisbane, QLD, Australia; ^5^Skaggs School of Pharmacy and Pharmaceutical Sciences, University of California, San Diego, La Jolla, CA, United States; ^6^Centre for Stem Cell Systems, University of Melbourne, Melbourne, VIC, Australia; ^7^Institute for Molecular Bioscience and IMB Centre for Inflammation and Disease Research, University of Queensland, Brisbane, QLD, Australia

**Keywords:** ScpC, PrtS, IL-8 protease, intracellular infection, *Streptococcus pyogenes*

## Abstract

*Streptococcus pyogenes* (Group A *Streptococcus*; GAS) commonly causes pharyngitis in children and adults, with severe invasive disease and immune sequelae being an infrequent consequence. The ability of GAS to invade the host and establish infection likely involves subversion of host immune defenses. However, the signaling pathways and innate immune responses of epithelial cells to GAS are not well-understood. In this study, we utilized RNAseq to characterize the inflammatory responses of primary human tonsil epithelial (TEpi) cells to infection with the laboratory-adapted M6 strain JRS4 and the M1T1 clinical isolate 5448. Both strains induced the expression of genes encoding a wide range of inflammatory mediators, including IL-8. Pathway analysis revealed differentially expressed genes between mock and JRS4- or 5448-infected TEpi cells were enriched in transcription factor networks that regulate IL-8 expression, such as AP-1, ATF-2, and NFAT. While JRS4 infection resulted in high levels of secreted IL-8, 5448 infection did not, suggesting that 5448 may post-transcriptionally dampen IL-8 production. Infection with 5448Δ*cepA*, an isogenic mutant lacking the IL-8 protease SpyCEP, resulted in IL-8 secretion levels comparable to JRS4 infection. Complementation of 5448Δ*cepA* and JRS4 with a plasmid encoding 5448-derived SpyCEP significantly reduced IL-8 secretion by TEpi cells. Our results suggest that intracellular infection with the pathogenic GAS M1T1 clone induces a strong pro-inflammatory response in primary tonsil epithelial cells, but modulates this host response by selectively degrading the neutrophil-recruiting chemokine IL-8 to benefit infection.

## Importance

Group A streptococcal pharyngitis places a significant burden on global health, with no protective vaccine currently available for human use. Despite more than 200 different identified GAS M types, GAS pharyngitis is disproportionately caused by the M1T1 strain in high income countries. An improved understanding of the host responses that mediate immunity to GAS infection will help facilitate the development of better strategies to combat GAS pharyngitis. In this study we investigated innate immune responses elicited during intracellular infection of primary human tonsil epithelial cells with the M1T1 strain 5448 at both the gene expression and protein level. We report that 5448 post-transcriptionally dampens host IL-8 responses during intracellular infection, and this effect is mediated by the serine protease SpyCEP. Our data reinforces the idea that the success of the M1T1 clone is due to its ability to modulate host inflammatory responses to benefit infection.

## Introduction

*Streptococcus pyogenes* (Group A *Streptococcus*; GAS) is the most common bacterial cause of acute pharyngitis (Bisno, 2001), with over 616 million incident cases of GAS pharyngitis predicted to occur worldwide each year (Ralph and Carapetis, 2013). GAS pharyngitis has also been associated with inflammatory complications such as scarlet fever (Wessels, 2016), chronic diseases including tonsillar hypertrophy and sleep apnea (Viciani et al., 2016), and the post-infectious sequelae including acute rheumatic fever (Gerber et al., 2009), poststreptococcal glomerulonephritis (Rodriguez-Iturbe and Musser, 2008) and guttate psoriasis (Gudjonsson et al., 2003). During pharyngitis, GAS colonizes the mucosal layers of the pharynx including the palatine tonsil epithelium, forming biofilms on the tonsillar surface, and inside tonsillar crypts, as well as invading the epithelium to survive intracellularly (Osterlund et al., 1997; Roberts et al., 2012). The ability of GAS to colonize the host and establish infection likely involves subversion of host immune defenses (Walker et al., 2014).

Epithelial cells are among the first cell types encountered by GAS during pharyngeal infection. During the early stages of infection, epithelial cells play important roles in barrier defense, secretion of antimicrobial peptides, and innate immune signaling (Soderholm et al., 2017). In response to GAS infection, immortalized epithelial cell lines derived from epithelial cell cancers, such as Detroit 562 (cancer of the pharynx), HEp-2 (HeLa derivative epithelial cells derived from the cervix), HaCaT (skin keratinocytes), and A549 (lung tissue derivative) cells have been shown to express and/or secrete a wide array of pro-inflammatory mediators, including chemokines (IL-8, CXCL2, CXCL3, CCL5/RANTES), cytokines (IL-1α, IL-1β, IL-2, IL-6, TNFα, TNFβ, IFN-γ, CSF2, IL-17A, IL-23), growth factors (IGF-II), and the eicosanoid prostaglandin E_2_ (Courtney et al., 1997; Wang et al., 1997; Nakagawa et al., 2004; Klenk et al., 2005, 2007; Tsai et al., 2006; Rizzo et al., 2013). In addition, GAS-infected Detroit 562 cells have been shown to secrete the chemokines CXCL9, CXCL10, and CXCL11 (Egesten et al., 2007; Linge et al., 2007). However, a potential limitation of these studies is that immortalized cell lines may not respond in the same way as primary tonsil epithelial cells to GAS infection. While the limited studies utilizing human tonsil explants have been fairly consistent with these cell line results (Agren et al., 1998; Wang et al., 2010; Bell et al., 2012), as these studies did not isolate epithelial cells from the tonsil explant tissue, it is difficult to elucidate the contribution of tonsil epithelial cells to these immune responses during GAS infection.

Pro-inflammatory gene expression during GAS infection of nonpharyngeal-derived immortalized epithelial cell lines has been shown to be driven by the activation of mitogen-activated protein kinases (MAPK) signaling pathways (extracellular signal-regulated kinase, ERK; Jun N-terminal protein kinase, JNK; and p38), as well as the transcription factors NF-κB and AP-1 (Medina et al., 2002; Tsai et al., 2006; Klenk et al., 2007; Persson et al., 2015; Chandrasekaran and Caparon, 2016). For example, exposure of HEp-2 cells to GAS strain A40 resulted in activation of the classical NF-κB pathway (Medina et al., 2002). Another study showed that inducible IL-6 and IL-8 production from HEp-2 cells upon infection with GAS strain M29588 was blocked by NF-κB and MAPK inhibitors (Tsai et al., 2006). Studies using the HEp-2 cell line and primary mouse bone marrow-derived macrophages have also shown that the magnitude of cytokine responses to GAS infection is often genotype (*emm*-type)-dependent, making these findings difficult to generalize for other *emm* types (Klenk et al., 2007; Dinis et al., 2014). A possible explanation for this observation is that certain GAS strains may be able to subvert host inflammatory responses during infection. However, the underlying GAS virulence factors and host-pathogen interactions leading to these differing cytokine responses are currently not well-defined.

The aim of this study was to identify, through the use of RNAseq and pathway analysis, key innate immune signaling responses and downstream biological effects that are initiated by primary human tonsil epithelial (TEpi) cells upon M1T1 GAS infection. This approach revealed transcription factor networks, including activator protein-1 (AP-1), activating transcription factor 2 (ATF-2), and nuclear factor of activated T cells (NFAT) pathways, as signaling hubs that control GAS-regulated IL-8 expression. Subsequent validation studies revealed that, whilst infection of TEpi cells with the laboratory-adapted GAS strain JRS4 induced strong IL-8 secretion, infection with the clinical M1T1 clone (strain 5448) did not, which we demonstrate to be dependent on the activity of the IL-8 protease SpyCEP. This study provides insight into the modulation of the tonsillar immune response during infection with M1T1 GAS strains, which may contribute to the success of this globally-disseminated human pathogen.

## Results

### Intracellular infection of TEpi cells with 5448 or JRS4 GAS strains induces the transcriptional upregulation of multiple pro-inflammatory pathways

**Figure 1:**
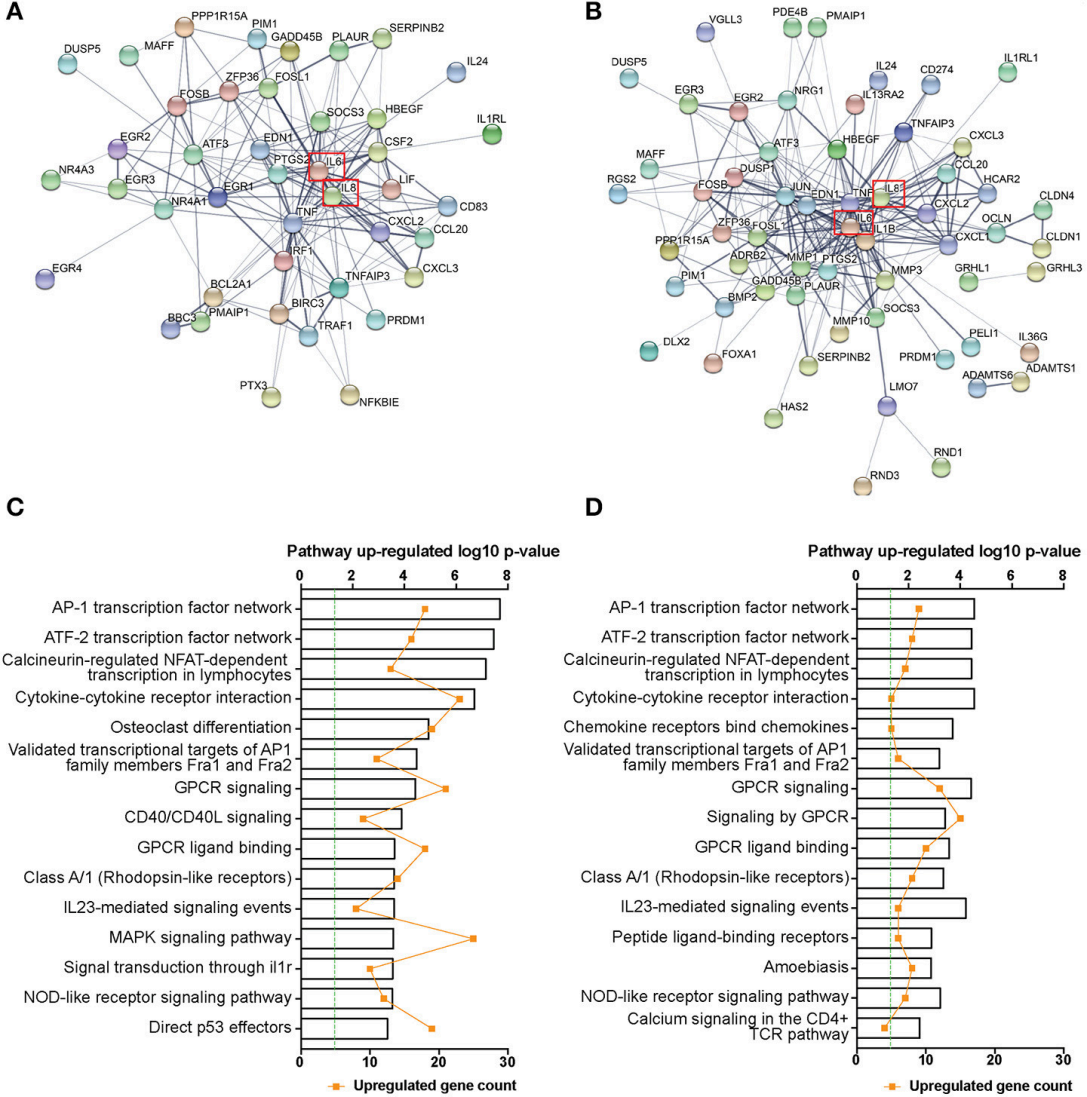
RNAseq transcriptome network and pathway enrichment of 5448- and JRS4 GAS-infected TEpi cells in comparison to mock cells. Protein-protein interaction network of the top 100 differentially expressed genes (at an adjusted *P* < 0.05) for **(A)** 5448-infected TEpi cells in comparison to mock TEpi cells and **(B)** JRS4-infected TEpi cells in comparison to mock TEpi cells, generated using STRINGdb (*http://string-db.org/*). IL-8 is highlighted (red box). Network edges show the confidence of interactions, where the line thickness indicates the strength of data support. Active interaction sources include textmining, experiments, databases, co-expression, neighborhood, gene fusion and co-occurrence. Minimum required interaction score of medium confidence (0.400). Non-protein coding genes and disconnected nodes are not shown. Node color is arbitrary. **(C)** Pathway over-representation analysis of all differentially expressed genes (adjusted *P* < 0.05, Log2FC >1 or <-1) for 5448 infected TEpi cells in comparison to mock cells and **(D)** JRS4-infected TEpi cells in comparison to mock cells was performed using *Innatedb.com*. Fold-change cutoff (±) 1.0 and *P*-value cutoff 0.05. Hypergeometric analysis algorithm was used, with Benjamini–Hochberg correction method. Top 15 up-regulated pathways shown. Green line indicates threshold for significance. Metadata from analysis results shown in Tables S1, S2.

Previous studies utilizing immortalized epithelial cell lines have shown an array of pro-inflammatory mediators are induced following GAS challenge (Courtney et al., 1997; Wang et al., 1997; Klenk et al., 2005, 2007; Tsai et al., 2006; Egesten et al., 2007; Linge et al., 2007), however, the response of primary tonsil epithelial cells to GAS infection has not been characterized. In addition, different GAS serotypes have previously been shown to induce epithelial inflammatory responses of differing magnitudes (Klenk et al., 2007; Persson et al., 2015). To investigate whether this variation exists in primary cells and to identify potential mechanisms, we performed RNAseq on TEpi cells infected with GAS strains JRS4 or 5448, for 6 h at a multiplicity of infection (MOI) of 5, an MOI where no significant difference in cell death was observed between the two strains and mock-treated cells (Figure S1). No difference in intracellular bacteria numbers or intracellular survival was observed between JRS4 and 5448 GAS strains (Figure S2). After 6 h infection of TEpi cells with 5448, 1109 genes were significantly up or down regulated in comparison to uninfected (mock) TEpi cells (adjusted *P* < 0.05, Log2 fold change >1 or <-1), and 153 genes were differentially expressed following infection with the JRS4 strain in comparison to mock cells. Due to the large difference in the number of differentially expressed genes induced between the two strains at this threshold, the top 100 most differentially expressed genes in JRS4-infected and 5448-infected TEpi cells in comparison to mock cells (Tables S1, S2) were used to construct predicted protein-protein interaction networks. Both strains resulted in the expression of an abundance of inflammatory mediators by the TEpi cells, and amongst these was a highly connected network of interacting proteins, with inflammatory mediators such as IL-8 and IL-6 at the core of the network (Figures 1A,B). Within these networks, interacting genes encoding proteins from several inflammatory pathways were identified. For 5448-infected TEpi cells, these genes included transcription factors and signaling proteins from the AP-1, ATF-2, and NFAT transcription factor networks (*FOSB, FOSL1, ATF3, EGR1, EGR2, EGR3, EGR4, DUSP5*). Cytokines (*TNF, IL6, CSF2, LIF*), chemokines (*IL8, CXCL2, CCL20, CXCL3*), and peptide hormones (*EDN1*) regulated by these pathways were also detected. Other interacting genes detected including those encoding components of the NF-κB pathway (*NFKBIE, TRAF1, BIRC3, TNFAIP3*), apoptosis (*BBC3, PMAIP1, BCL2A1, IRF1*), and MAPK pathway (*GADD45B, PIM1*) (Figure 1A). For JRS4-infected TEpi cells, interacting genes were also detected for AP-1, ATF-2, and NFAT transcription factor networks, for transcription factors/signaling proteins (*JUN, FOSB, FOSL1, ATF3, DUSP1, DUSP5, EGR2, EGR3*), and inflammatory transcriptional targets of these pathways (*TNF, IL8, IL6, IL1B, MMP1, CXCL1, CXCL2, CXCL3, CCL20*). Interacting genes were also detected for tight junctions and epithelial barrier integrity (*OCLN, CLDN4, CLDN1, GRHL1, GRHL3*; Figure 1B).

Pathway enrichment tests identified a role for the AP-1, ATF-2, and NFAT transcription factor networks and GPCR signaling in TEpi cell responses to 5448 infection (Figure 1C). These pathways are known to drive the transcription of inflammatory cytokines such as *IL6, TNF*, and *IL8* following activation (Eliopoulos et al., 1999; Jundi and Greene, 2015; Kaunisto et al., 2015). Fewer genes were significantly differentially expressed following JRS4 infection, but many of the induced pathways were common to 5448-infected TEpi cells (Figure 1D). This indicated that 5448 induces a stronger TEpi cell inflammatory response, at the transcriptional level, compared to JRS4 infection. No pathways were enriched and significantly downregulated following either 5448 or JRS4 infection. Metadata from group comparisons is shown in Tables S1, S2.

### 5448 GAS infection activates inflammatory pathways which lead to inducible *IL8* expression

**Figure 2:**
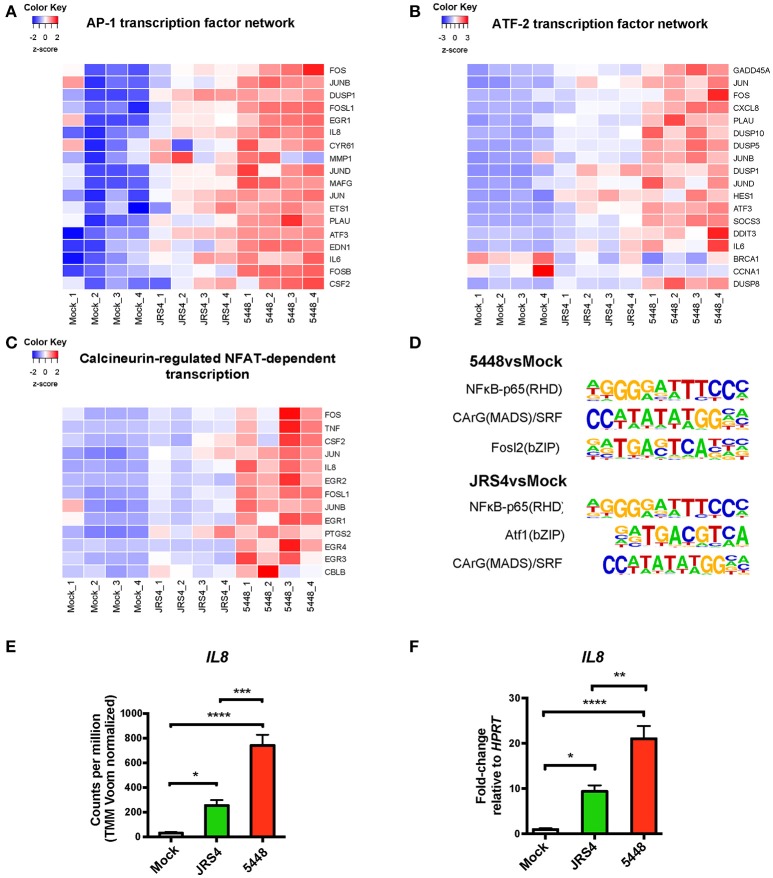
Regulated IL-8 expression in 5448 and JRS4 GAS-infected TEpi cells in comparison to mock cells. Heat maps of differentially expressed genes from top pathways enriched by *Innatedb.com* that induce IL-8 gene transcription. **(A)** AP1 transcription factor network, **(B)** ATF-2 transcription factor network, and **(C)** Calcineurin-regulated NFAT-dependent transcription. **(D)** Known transcription factor motifs enriched from top 400 differentially expressed genes for 5448-infected TEpi cells and JRS4-infected TEpi cells using HOMER motif analysis. 5448vsmock: NF-κB-p65 Rel homology domain (RHD) target sequences with motif = 42. CArG(MADS-domain)/Serum response factor (SRF) target sequences with motif = 21. Fosl2/basic Leucine Zipper (bZIP) target sequences with motif = 21. JRS4vsmock: NF-κB-p65(RHD) target sequences with motif = 38. Atf1/(bZIP) target sequences with motif = 58. CArG (MADS) target sequences with motif = 19. **(E)** RNAseq *IL8* read counts. **(F)** qPCR of IL-8 mRNA levels, normalized to *HPRT*. Data in **(E,F)** (mean ± s.e.m.) are combined from at least three independent experiments performed in triplicate and analyzed by one-way ANOVA with Tukey's post-test. ^*^P < 0.05; ^**^P < 0.01; ^***^P < 0.001; ^****^P < 0.0001.

The expression of genes contributing to each pathway was visualized using a heat map. The differences in amplitude of response between 5448- and JRS4-infected TEpi cells was consistently observed across each up-regulated network, and was apparent for the AP-1, ATF-2, and NFAT transcription factor networks (Figures 2A–C). Euclidean distance of samples showed highest similarity between replicates from each infection condition, with AP-1 and ATF-2 networks separating 5448-infected TEpi cells from mock-treated or JRS4-infected cells (Figures 2A,B). For JRS4-infected TEpi cells, most differentially expressed genes are at a lower expression level than following 5448-infection. A protein-protein interaction network derived from the top 100 most differentially expressed genes in 5448-infected TEpi cells compared to JRS4-infected cells, revealed gene clusters of transcription factors and cytokines/chemokines from the AP-1 and NFAT transcription factor networks (*FOSB, EGR1, EGR2, EGR3, EGR4, TNF, LIF, CXCL2, CCL20*). Pathway analysis also detected an enrichment of differentially expressed genes in the NFAT and AP-1 networks (Figure S3, metadata shown in Table S3).

Transcription factor motif enrichment using HOMER motif analysis software was performed on the top 400 differentially expressed genes for 5448-infected and JRS4-infected TEpi cells (Heinz et al., 2010). Enrichment of known motifs in differentially expressed genes identified enrichment of motifs for NF-κB (Oeckinghaus and Ghosh, 2009) and serum response factor (SRF) (Spencer and Misra, 1996) transcription factors in both JRS4- and 5448-infected TEpi cells (Figure 2D). Forty-two 5448-induced genes contained the NF-κB motif (enrichment *P* < 0.001), and this motif was also highly represented in genes responding to JRS4 infection (38 differentially expressed genes contained the motif, enrichment *P* < 0.01). Similarly, 21 differentially expressed genes contained the SRF motif following 5448 infection (enrichment *P* < 0.001), and 19 differentially expressed genes (*P* < 0.01) contained the SRF motif following JRS4 infection. The basic Leucine Zipper Domain (bZIP) motif (Ellenberger et al., 1992) was also enriched after infection with either GAS strains [21 differentially expressed genes following 5448 infection (enrichment *P* < 0.001); 58 differentially expressed genes following JRS4 infection (enrichment *P* < 0.001)]. The bZIP recognition motif is bound by multiple transcription factors including ATF-1 and Fosl2/Fra-2, which are members of the AP-1 and ATF-2 transcription factor networks (Hai and Curran, 1991). This analysis indicates that the same signaling pathways are engaged by both strains, but the amplitude of signaling is reduced in JRS4-infected TEpi cells, perhaps by abrogated feedforward amplification via cytokine signaling.

### 5448 Suppresses levels of secreted IL-8 in primary human tonsil epithelial cells

**Figure 3:**
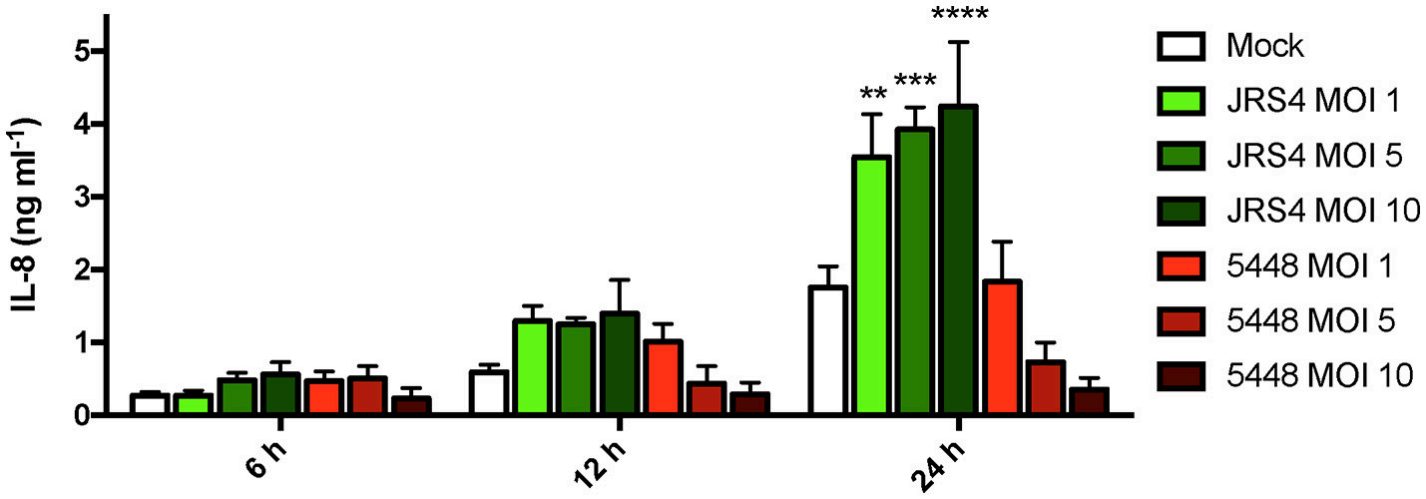
5448-infected TEpi cells show reduced levels of IL-8 detected. IL-8 produced by TEpi cells intracellularly infected with GAS strains 5448 and JRS4, as measured by ELISA. Data are plotted as the mean ± s.e.m. and are combined from three independent experiments performed in triplicate and analyzed by two-way ANOVA with Tukey's post-test. Significance is shown relative to mock at each timepoint. ^**^P < 0.01; ^***^P < 0.001; ^****^P < 0.0001.

The RNAseq highlighted increased *IL8* mRNA expression following both JRS4 and 5448 infection, with 5448-infected TEpi cells inducing *IL8* mRNA to levels that were 23-fold higher than mock cells, and 2.9-fold higher than JRS4-infected cells (Figure 2E). These strain differences in inducing *IL8* were confirmed by qRT-PCR analysis of gene expression at 6 h post-infection; in these experiments, *IL8* mRNA levels were 21-fold higher in 5448-infected TEpi cells than in mock cells, and 2.2-fold higher than JRS4-infected cells (Figure 2F). To determine whether the increased *IL8* transcription following GAS infection resulted in increased IL-8 production by these cells, we next quantified IL-8 secretion by TEpi cells. TEpi cells were cultured to confluent monolayers, infected with GAS strains JRS4 and 5448 at multiple MOIs (1, 5, and 10), and IL-8 levels quantified by ELISA at 6, 12, and 24 h post-infection (Figure 3). Surprisingly, while the IL-8 response to JRS4 infection increased in a time- and MOI-dependent manner, the IL-8 response to 5448 infection did not increase above that of mock cells, and decreased with increasing MOI. In order to ensure the differences in cytokine secretion were not due to differences in cell death induced by the two GAS strains, tonsil cell death was measured by quantifying the lactate dehydrogenase levels released by TEpi cells following with each strain (Figure S1). Both strains induced TEpi cell death at 24 h, however the level of cell death induced by JRS4 and 5448 was not significantly different between the two strains.

### SpyCEP degrades IL-8 during infection of TEpi cells

**Figure 4:**
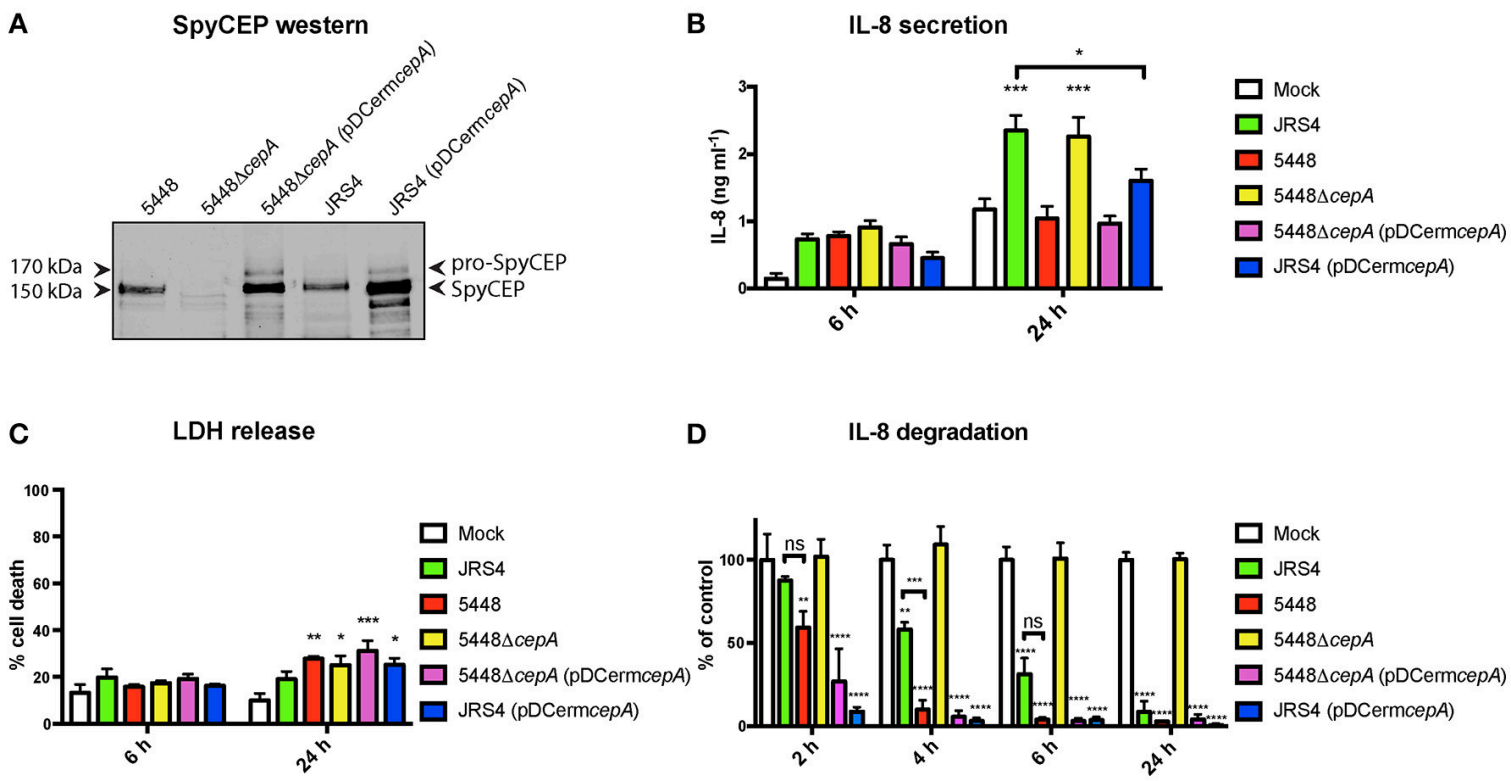
SpyCEP expression by 5448 results in reduced IL-8 secretion by TEpi cells during GAS intracellular infection. **(A)** Western blot analysis of cell wall extracts for detection of SpyCEP. Equivalent protein concentrations were loaded for 5448, JRS4, and 5448Δ*cepA* (100 μg), whilst 5 μg was loaded for 5448Δ*cepA* (pDCerm*cepA*) and JRS4 (pDCerm*cepA*). **(B)** IL-8 present in supernatants due to secretion by TEpi cells following GAS intracellular infection at 6 and 24 h post-infection, measured by ELISA. **(C)** Cell death measured by percentage of LDH released from TEpi cells at 6 and 24 h post-infection. **(D)** GAS strains were incubated with recombinant IL-8 at 37°C. SpyCEP activity correlates with IL-8 degradation as assessed by ELISA. For **(B–D)**, data are plotted as the mean ± s.e.m. and represent three independent experiments performed in triplicate and analyzed by two-way ANOVA with Tukey's post-test. Significance shown is relative to mock at each timepoint, unless otherwise indicated. ^*^P < 0.05; ^**^P < 0.01; ^***^P < 0.001; ^****^P < 0.0001.

We considered the possibility that the decrease in levels of secreted IL-8 in 5448-infected TEpi cells may relate to proteolytic degradation of this key neutrophil-recruiting chemokine. SpyCEP is a 170 kDa serine protease expressed on the surface of GAS, which proteolytically cleaves and abrogates the activities of the human chemokines CXCL1, CXCL2, CXCL6, and CXCL8/IL-8, impairing neutrophil recruitment to the site of infection (Edwards et al., 2005; Hidalgo-Grass et al., 2006; Sumby et al., 2008; Zinkernagel et al., 2008; Turner et al., 2009). In order to investigate the impact of SpyCEP expression on IL-8 secretion during intracellular GAS infection of TEpi cells, the GAS strains 5448, JRS4, and 5448 SpyCEP knockout (5448Δ*cepA*) were utilized. A plasmid expressing 5448-derived SpyCEP (pDCerm*cepA*) was introduced into both JRS4 and 5448Δ*cepA* strains to confirm SpyCEP specificity for the observed phenotype. Comparison of the genome sequences of JRS4 (Port et al., 2015) and 5448 (Fiebig et al., 2015) revealed that both GAS strains contained the SpyCEP encoding gene *cepA*, that would encode proteins with 99% amino acid sequence identity, and conserve the catalytic triad residues required for serine-protease activity. However, several amino acid differences between 5448 and JRS4 were observed in the N-terminal region of SpyCEP (Figure S4), suggesting that there might be differences in secretion and/or cell surface localization of this protein between 5448 and JRS4. To determine whether both GAS strains produce surface-anchored SpyCEP, western blot analysis was employed to detect SpyCEP expression in the cell wall fraction of GAS strains (Figure 4A). Equivalent protein concentrations were loaded for 5448, JRS4, and 5448Δ*cepA* strains. A protein band at the predicted molecular weight of the surface-bound cleaved form of SpyCEP (150 kDa) was detected for both the 5448 and JRS4 strains. This protein band was not detected for 5448Δ*cepA*. Detectable levels of the 170 kDa protein, corresponding to the uncleaved form of SpyCEP, were observed following introduction of *cepA* on a plasmid in strains 5448Δ*cepA* (pDCerm*cepA*) and JRS4 (pDCerm*cepA*).

The levels of IL-8 secreted by TEpi cells following intracellular infection with GAS strains (MOI of 5) at 6 and 24 h post-infection were measured by ELISA. At 6 h post-infection, IL-8 secretion was not significantly elevated for all infections compared to mock cells (Figure 4B). At 24 h post-infection, JSR4 induced a significant increase in levels of secreted IL-8 compared to mock TEpi cells, whilst 5448 did not induce an IL-8 response. 5448Δ*cepA* induced an IL-8 response similar to JRS4 levels, and the complemented 5448Δ*cepA* (pDCerm*cepA*) IL-8 response was similar to 5448 levels. Infection with JRS4 expressing *cepA* (pDCerm*cepA*) significantly reduced the IL-8 response of TEpi cells in comparison to JRS4-infected cells at 24 h post-infection (*P* < 0.05; Figure 4B). To determine whether the presence or absence of SpyCEP expression impacts the level of TEpi cell death, lactate dehydrogenase release was used to quantify cell death following GAS intracellular infection. No significant difference in cell death was observed between cells infected with 5448, 5448Δ*cepA*, 5448Δ*cepA* (pDCerm*cepA*), JRS4, or JRS4 (pDCerm*cepA*) (Figure 4C). To confirm that the effect of SpyCEP on IL-8 production was due to its ability to proteolytically degrade IL-8, we next quantified IL-8 levels by ELISA following incubation of recombinant IL-8 with each GAS strain over a time course. Both JRS4 and 5448 strains degraded IL-8, however 5448 degraded IL-8 more efficiently than JRS4 after 4 h incubation (*p* < 0.001). IL-8 degradation was not observed following incubation with 5448Δ*cepA*, whilst the complemented strains 5448Δ*cepA* (pDCerm*cepA*) and JRS4 (pDCerm*cepA*) exhibited enhanced IL-8 degradation (Figure 4D).

## Discussion

Whilst GAS is the most common cause of bacterial pharyngitis, our understanding of the host innate immune responses during GAS colonization of the tonsil is surprisingly limited. Given that the M1T1 strain disproportionately causes GAS pharyngitis in high income countries, a better understanding of the host responses that mediate immunity to M1T1 GAS infection could lead to the development of better therapeutic and preventative strategies to combat GAS pharyngitis. Moreover, understanding the fundamental aspects of host innate immunity is likely to provide additional insights into the pathology of other bacterial pathogens that colonize the human pharynx.

Here we demonstrate that the highly successful and globally-disseminated M1T1 GAS clone (strain 5448) induces a strong pro-inflammatory transcriptional response, yet degrades IL-8 post-transcriptionally through expression of a protease. IL-8 plays an important role in the recruitment of neutrophils during infection (Tsai et al., 2006), which is likely an important protective mechanism elicited by epithelial cells during GAS pharyngitis (Persson et al., 2015). IL-8 was one of the most differentially expressed chemokines at the level of mRNA expression following intracellular infection with both 5448 and a laboratory-adapted strain (JRS4). In contrast, whilst JRS4 infection resulted in the secretion of IL-8 at levels significantly higher than mock TEpi cells, 5448 infection did not result in increased levels of secreted IL-8 beyond that observed for mock TEpi cells. This lack of response in 5448-infected cells was found to be dependent on the expression of the GAS virulence factor SpyCEP. The function of SpyCEP has been defined by previous studies as a serine protease that cleaves the human chemokines CXCL1, CXCL2, CXCL6, and CXCL8/IL-8, abrogating their biological activity, and enabling resistance of GAS to phagocytic clearance (Edwards et al., 2005; Sumby et al., 2008; Zinkernagel et al., 2008; Turner et al., 2009). SpyCEP expression by the 5448 strain has also been reported to decrease the adherence and invasion of GAS into epithelial cells, as well as impair biofilm formation, suggesting that SpyCEP expression may modulate colonization and GAS-host cell interactions (Andreoni et al., 2014). This study highlights the important role played by SpyCEP during infection, by demonstrating that different GAS strains (5448 and JRS4) exhibit varying levels of IL-8 degradative activity, resulting in varying levels of IL-8 secretion during intracellular infection of primary epithelial cells.

SpyCEP is a highly conserved virulence factor across GAS M types (Sumby et al., 2008); however different GAS strains have been shown to exhibit varying levels of SpyCEP activity as a result of *covRS* mutation (Turner et al., 2009). Our results show that whilst both JRS4 and 5448 GAS strains express SpyCEP at similar levels (Figure 4A), JRS4 showed reduced efficiency in cleaving human recombinant IL-8 (Figure 4D). This reduced SpyCEP activity could explain the IL-8 secretion detected during JRS4 intracellular infection of TEpi cells (Figure 4B). In contrast, 5448 efficiently cleaved human recombinant IL-8, supporting the hypothesis that 5448 SpyCEP degrades IL-8 during TEpi cell infection. The cause of this difference in SpyCEP activity or protein levels is unknown. Amino acid sequence alignment shows the 3 amino acids encoding the catalytic triad responsible for serine protease activity and the residues around this region are conserved, while the N-terminal region of *cepA* shows some variability in amino acids for JRS4, as compared to 5448. It is not known what role these specific residues play in the protease activity of SpyCEP. However, the N-terminal region is an important component of the protease, as it encodes the 20 kDa fragment that is cleaved from the 170 kDa full length SpyCEP resulting in protease activity (Zingaretti et al., 2010). This 20 kDa fragment then associates with the 150 kDa cell-wall anchored portion of SpyCEP, with both fragments required for protease activity (Zingaretti et al., 2010). Previous studies have also shown that different GAS M types induce differing levels of secreted IL-8 by immortalized epithelial cells (Klenk et al., 2007; Persson et al., 2015); differences between GAS M types in the capacity of SpyCEP to cleave IL-8 could explain this observation.

Whilst this study has shown that 5448-derived SpyCEP induces IL-8 degradation during intracellular GAS infection, the cellular location where SpyCEP encounters IL-8 is not known. IL-8 secretory pathways are not well-characterized in epithelial cells; however, the current understanding is that intracellular IL-8 is trafficked in secretory vesicles (Stanley and Lacy, 2010). Some studies have reported cytoplasmic IL-8 present in the epithelial cells from human tissue biopsies (Murphy et al., 2005; Ulukus et al., 2009) and in *Chlamydia trachomatis* infected HeLa cells (Buchholz and Stephens, 2006), suggesting that there may be another uncharacterized mechanism by which epithelial cells secrete IL-8. Cytoplasmic IL-8 would enable SpyCEP direct access to cleave IL-8 during intracellular infection. It is also plausible that SpyCEP degradation of IL-8 does not occur intracellularly. SpyCEP is cell wall anchored in the strains utilized in this study, however, SpyCEP secretion and extracellular release of SpyCEP during infection could also contribute to the IL-8 degradation observed.

Previous studies have demonstrated that, during infection of immortalized epithelial cells, GAS activates the AP-1, NF-κB, and MAPK pathways, leading to increased expression and secretion of key cytokines and chemokines, particularly IL-8 and IL-6 (Medina et al., 2002; Tsai et al., 2006; Klenk et al., 2007; Persson et al., 2015; Chandrasekaran and Caparon, 2016). These inflammatory responses serve as an early signaling system to activate and recruit immune cells to the site of infection, thereby helping combat bacterial infection. The findings from this study show that both 5448 and JRS4 GAS strains induce the expression of inflammatory mediators enriched in the AP-1, ATF-2, and NFAT pathways, all of which are linked to the production of IL-8 (Eliopoulos et al., 1999; Jundi and Greene, 2015; Kaunisto et al., 2015). Both NFAT and ATF-2 are able to bind cooperatively with transcription factors of the AP-1 (Fos/Jun) family to composite NFAT:AP-1 or ATF-2:AP-1 sites. These sites are found in the regulatory regions of genes that are inducibly transcribed during immune responses, including cytokines and other genes critical to immune signaling and activation (Rao et al., 1997). HOMER analysis detected the enrichment of motifs in the promoters of differentially expressed genes for NF-κB and the MAPK-activated transcription factor SRF, indicating that these pathways are likely upstream of AP-1/ATF-2/NFAT-mediated gene expression, as detected by RNAseq at 6 h post-infection. 5448 and JRS4 GAS infection also activated G protein-coupled receptor (GPCR) signaling (Figures 1C,D), which is another pathway that may regulate inflammatory responses during GAS infection (Bestebroer et al., 2010). GPCRs play a critical role in modulating the expression or activity of transcription factors through complex signaling networks, which includes NF-κB, AP-1 MAPK, NFAT, SRF, and ATF-2 (Reichle, 2010; Sun and Ye, 2012). Inflammatory mediators such as chemokines and complement proteins can stimulate the activation of specific GPCRs, leading to the expression of cytokines such as IL-6, IL-8, TNF-α, and IL-1β (Ye, 2001; Lodowski and Palczewski, 2009). It should also be noted that 5448-infection was found to induce an inflammatory transcriptional response in TEpi cells of a much higher magnitude than during JRS4-infection. Previous studies have also shown that the magnitude of host inflammatory mediators in response to GAS is *emm*-type dependent (Klenk et al., 2007; Dinis et al., 2014; Persson et al., 2015). As 5448 is an M1T1 clinical GAS isolate and JRS4 an M6 laboratory adapted isolate, there is likely a difference in the virulence factor expression profiles of these strains during intracellular infection, which may be driving the large difference in TEpi cell transcriptional responses detected. The virulence factors responsible for this, however, remain to be defined.

In summary, we have shown the pathogenic M1T1 strain 5448 induces robust changes in pro-inflammatory gene expression following intracellular infection of primary human tonsil epithelial cells, and degrades host-protective neutrophil-recruiting IL-8, which is driven by the activity of the GAS serine protease SpyCEP. This is an exciting and potentially novel mechanism by which a bacterial pathogen modulates the host inflammatory response and supports the hypothesis that the high burden of pharyngitis caused by M1T1 GAS is due to its ability to modulate host inflammatory responses that are triggered by infection.

## Materials and methods

### Bacterial strains and growth conditions

GAS M1T1 strain 5448 was originally isolated from a patient with necrotizing fasciitis and toxic shock syndrome (Chatellier et al., 2000). JRS4 is a streptomycin-resistant derivative of D471, a rheumatic fever-associated isolate (Norgren et al., 1989). 5448Δ*cepA* and 5448Δ*cepA* complemented with pDCerm*cepA* are described by Zinkernagel et al. (2008). JRS4 (pDCerm*cepA*) was generated by isolating total DNA from 5448Δ*cepA* (pDCerm*cepA*), using Wizard® Plus SV miniprep DNA Purification System (Promega, A1460). Plasmid DNA was then purified using the Qiagen DNeasy blood and tissue kit (Cat. # 69504). The pDCerm*cepA* plasmid was then transformed into *E. coli* MC1061, and purified by Qiagen Plasmid PlusMidi Kit (Cat. #12943). JRS4 was electroporated with 2 μg of pDCerm*cepA*, before being plated onto Todd-Hewitt agar supplemented with 0.2% yeast extract (THY) and erythromycin (4 μg/ml) for 48 h at 37°C. GAS strains were grown in Todd-Hewitt broth supplemented with 0.2% yeast extract (THY) at 37°C. Where appropriate, erythromycin was supplemented at 4 μg/ml.

### Human primary tonsil epithelial cells

Human primary tonsil epithelial (TEpi) cells were purchased from Sciencell Research Laboratories, California (Cat. #2560). TEpi cells consist of a heterogeneous population of actively differentiating stratified squamous epithelial cells and reticulated epithelial cells isolated from the tonsil explants of an individual donor (Lot. No. 10474, 10 y.o. male, caucasian). TEpi cells were cultured at 37°C under a 5% CO_2_/20% O_2_ atmosphere in Tonsil Epithelial Cell Medium (Sciencell, Cat. #2561) supplemented with 1% Tonsil Epithelial Cell Growth Supplement (Sciencell, Cat. #2562). TEpi cells were cultured on poly-L-lysine coated tissue culture vessels (Greiner Bio-one). At 95% confluency, cells were trypsinized and handled according to instructions provided by the manufacturer. TEpi cells were cultured for several weeks with media changed every 2–3 days, and utilized for experiments at passage 4.

### Intracellular infection assays

TEpi cells were seeded at a density of ~2 × 10^4^ viable cells per well in 24-well tissue culture plates or 12 × 10^4^ viable cells per well in 6 well plates coated with poly-L-lysine. Cells were grown at 37°C under a 5% CO_2_/20% O_2_ until they formed a confluent monolayer. Immediately prior to infection, the TEpi medium was removed, and replaced with fresh TEpi medium. GAS strains were grown to early stationary phase (OD_600_ = 1.2 for 5448 and OD_600_ = 1.6 for JRS4), re-suspended in cell culture medium, and then added to cell monolayers at the required multiplicity of infection (MOI). For the cytotoxicity assay, MOIs of 1, 5, and 10 were utilized, whilst for all other experiments an MOI of 5 was used. Controls included for each experiment were cells not exposed to bacteria (mock). At 2 h post-infection, the media was aspirated and cells were washed twice with fresh media, before TEpi media containing 100 μg/ml of gentamicin was added to each well for the duration of the experiment to kill extracellular GAS (Barnett et al., 2013). For each strain, colony forming unit (CFU) assays were performed following infection to retrospectively determine MOI. Bacterial cultures were serially diluted in PBS, before being spotted in triplicate onto THY agar plates and left to incubate for 24 h at 37°C. Bacterial colony numbers were then counted and the average CFU/ml calculated.

### Cytotoxicity assay

Cell death following GAS infection was quantified by measuring lactate dehydrogenase (LDH) release from cell supernatants, using CytoTox96® Non-Radioactive Cytotoxicity Assay (Promega, G1781), as per the manufacturer's instructions. Cells lysed in 0.2% Triton-X 100/1 × PBS were included as a positive control for 100% cell death, for each condition at each time point. Sample absorbance was measured by spectrophotometer (SpectraMax Plus 384, Softmax Pro 5.4 software) at an absorbance of 490 nm. Sample readings were analyzed by Prism 7 software and divided by the positive control for cell lysis to give a percentage of total cell death for each sample.

### RNA extraction

Tissue culture medium was aspirated from TEpi cell monolayers and RNA lysis buffer [Buffer RLT (Qiagen) plus 0.1 M β-mercaptoethanol] added to each well. Cells were scraped into the buffer using a cell-scraper, and passed through a QIAshredder homogenizer column (Qiagen, Cat. #79654) by centrifugation. RNA was then isolated using the RNeasy Mini Kit (Qiagen, Cat. #74104), according to the manufacturer's instructions. Absorbance of RNA samples was measured at 260 nm using a ND1000 Nanodrop (Biolab), and RNA concentration (μg/ml) was determined by the A_260_ reading: A_260_ = 1.0 is equivalent to 40 μg/ml RNA. RNA integrity of each RNA sample was confirmed by Agilent 2100 BioAnalyzer analysis (RIN score of > 7.0).

### RNA sequencing and bioinformatics analyses

Confluent 6-well plates of TEpi cells were infected with JRS4 or 5448, as described above, at an MOI of 5 for 6 h. Mock-treated cells were included as a control. RNA from four biological replicates was extracted, as described above. RNA-Seq libraries were generated using the Illumina TruSeq Stranded mRNA LT Sample Prep Kit (Illumina, RS-122-2101/RS-122-2102), according to the standard manufacturer's protocol (Illumina, 15031047 Rev. E October 2013). Total RNA was enriched for mRNA using an oligo-dT bead isolation method. The enriched mRNA was then subjected to a heat fragmentation step aimed at producing fragments between 120 and 210 bp (average 155 bp). cDNA was synthesized from the fragmented RNA using SuperScript II Reverse Transcriptase (Invitrogen, Catalog no. 18064014) and random primers. The resulting cDNA was converted into dsDNA in the presence of dUTP to prevent subsequent amplification of the second strand and thus maintaining the “strandedness” of the library. Following 3′ adenylation and adaptor ligation, libraries were subjected to 15 cycles of PCR to produce libraries ready for sequencing. The libraries were quantified on the Agilent BioAnalyzer 2100 using the High Sensitivity DNA Kit (Agilent, 5067-4626). Libraries were pooled in equimolar ratios, and the pool was quantified by qPCR using the KAPA Library Quantification Kit—Illumina/Universal (KAPA Biosystems, Part no. KK4824) in combination with the Life Technologies Viia 7 real time PCR instrument. Library preparation was performed at the Institute for Molecular Bioscience Sequencing Facility (University of Queensland).

Sequencing was performed using the Illumina NextSeq500 (NextSeq control software v2.0.2.1/Real Time Analysis v2.4.11). The library pool was diluted and denatured according to the standard NextSeq protocol (Illumina, Document # 15048776 v02), and sequenced to generate single-end 76 bp reads using a 75 cycle NextSeq500/550 High Output reagent Kit v2 (Illumina, FC-404-2005). After sequencing, fastq files were generated using bcl2fastq2 (v2.17.1.14). Sequencing was performed at the Institute for Molecular Bioscience Sequencing Facility (University of Queensland).

Quality-checked RNAseq FASTQ libraries were aligned to the reference Human genome (hg19) using the Subread (Liao et al., 2013) library version 1.18 for the R package for statistical computing version 3.2.1, with default mapping parameters. Reads were summarized and annotated to Ensembl version 69 transcripts and genes using the featureCounts function of Subread. A known batch effect in the post-normalized log2 expression data was successfully corrected for using the removeBatchEffect function of limma (Ritchie et al., 2015) version 3.26.8. Batch-corrected RPKM (Reads Per Kilobase of transcripts per Million mapped reads) and TMM normalized, log2 transformed expression data is available in Stemformatics (Wells et al., 2013) at *http://www.stemformatics.org/datasets/view/6991*. Raw data and count tables have been deposited to NCBI's Gene Expression Omnibus (GEO) (Edgar et al., 2002) under accession GSE107001.

Differential expression analysis was separately performed using R version 3.2.2 using the limma library version 3.26.8. The known batch effect was specified as part of the linear model and factored into the analysis. Genes were tested for differential expression if they displayed one count per million in all replicates of at least one phenotypic group. We used limma's Voom function to modify linear model fitting parameters based on the mean variance trend of counts observed. Input counts were TMM (Oshlack et al., 2010; Robinson and Oshlack, 2010) normalized and median-centered in addition to the log2 and count-per-million transforms applied by Voom. For downstream analyses, genes were considered differentially expressed with a Benjamini–Hochberg adjusted *p* < 0.05 and a Log2FC >1 or < −1.

### RNAseq interaction networks and pathway analysis

Protein-protein interaction networks from the top 100 differentially expressed genes (at an adjusted *P* < 0.05) from each group comparison (5448-infected TEpi cells vs. mock cells, JRS4-infected TEPi cells vs. mock cells, and 5448-infected vs. JRS4 infected TEPi cells) were constructed using STRINGdb.com (Szklarczyk et al., 2017). Network edges show the confidence of interactions, where the line thickness indicates the strength of data support. Active interaction sources include textmining, experiments, databases, co-expression, neighborhood, gene fusion, and co-occurrence. Minimum required interaction score of medium confidence (0.400). Pathway over-representation analysis of all differentially expressed genes (adjusted *P* < 0.05, Log2FC >1 or <-1) for 5448-infected TEpi cells in comparison to mock cells and JRS4-infected TEpi cells in comparison to mock cells was performed using InnateDB.com (Breuer et al., 2013). Hypergeometric analysis algorithm was used, with Benjamini–Hochberg correction method. HOMER motif analysis software was used to identify enriched transcription factor recognition motifs within the regulatory regions of the top 400 differentially expressed genes for 5448-infected tonsil epithelial cells and JRS4-infected TEpi cells (Heinz et al., 2010). Motif finding parameters: motifs of a length of 8, 10, or 12 bases from −300 to +50 bases relative to the transcription start site, with 2 mismatches allowed in global optimization phase. The background gene list used comprised of all gene IDs detected by RNAseq. Heat maps were drawn using R (version 3.3.2) gplots package, using RNAseq data available at NCBI's Gene Expression Omnibus (GEO) (Edgar et al., 2002), under accession GSE107001.

### Real-time qPCR

cDNAs were synthesized from extracted RNA using the Superscript® First-Strand Synthesis System (Thermofisher scientific, Cat. #18080051), as per the manufacturer's instructions. cDNA was diluted to 1 ng/μl in RNase-free water and used for qPCR. The relative expression of target genes was determined using the Applied Biosystems (ABI) ViiA^TM^ 7 Real-Time PCR System. A 15 μl reaction mixture was prepared with 2 × SYBR Green PCR Master Mix (Qiagen) (7.5 μl), 200 nm of forward and reverse primers, and 3 μl of diluted cDNA. cDNA and reagent master mixes (primer pair and SYBR Green) were automatically loaded into the 384-well plate using the Eppendorf epMotion 5075 robot and Eppendorf epBlue software. All cDNA samples were run in triplicate in a 384-well plate. Primers designed to amplify *IL8* were as follows: Forward (5′ – 3′) GGAGAAGTTTTTGAAGAGGGCTGA, and Reverse (5′ – 3′): TGCTTGAAGTTTCACTGGCATCTT. Standard qPCR cycles were used (10 min at 95°C step for polymerase activation, followed by 40 cycles of two-step thermal cyclic PCR at 95°C for 15 s and 60°C for 1 min). mRNA levels for specific genes were quantified relative to expression of the house-keeping gene hypoxanthine-guanine phosphoribosyltransferase (*HPRT*). Primers designed to amplify HPRT were as follows: Forward (5′ – 3′): TCAGGCAGTATAATCCAAAGATGGT, and Reverse (5′ – 3′): AGTCTGGCTTATATCCAACACTTCG. qPCR data was analyzed using QuantStudio^TM^ Software V1.2 and GraphPad Prism 6. The following equation was used to calculate relative gene expression: Gene/house-keeping gene = 2^−ΔCt^; where ΔCt = gene_Ct_–house-keeping gene_Ct_ (the difference in cycle thresholds).

### IL-8 ELISA

TEpi cells were infected with GAS as described above. At each time point post-infection, the media was removed from each well and centrifuged at 13,000 × *g* for 5 min at 4°C. The pellet was then discarded and the supernatant stored at −80°C. IL-8 protein levels were measured by ELISA according to manufacturer's directions (BD Biosciences OptEIA™, Cat. # 555244) using 96 well-microplates (Greiner Bio-One, Cat. # 655001). O-phenylenediamine dihydrochloride (Thermo Scientific Pierce, Cat. # 34006) was used as a substrate for horseradish peroxidase. Sample absorbance was measured by spectrophotometer (SpectraMax Plus 384, Softmax Pro 5.4 software) at 492 nm, with λ correction at 570 nm. Sample readings were analyzed using Prism 7 software, and protein concentrations were interpolated by linear regression of samples against a known standard curve of human IL-8.

### GAS cell wall fractionation and western blot analysis

The fractionation protocol was adapted from Sumby et al. (2008). GAS strains were grown to mid-exponential phase (OD_600_ = 0.45) in THY containing 28 μm E-64 (Sigma-Aldrich, E3132), before being pelleted by centrifugation at 8,000 × *g* for 20 min at 4°C. The bacterial pellet was washed twice with TE buffer (50 mM Tris-HCl, 1 mM EDTA; pH 8.0), supplemented with 1 mM PMSF (Sigma-Aldrich, P7626). The bacterial pellet was then resuspended in cold TE-sucrose buffer (50 mM Tris-HCl, 1 mM EDTA, 20% w/v sucrose; pH 8.0), supplemented with 5 mg lysozyme (Amresco, Cat. #0663), 200 U mutanolysin (Sigma-Aldrich, M9901), and protease inhibitor cocktail (Roche, Cat. #1187358). Samples were incubated for 2 h at 37°C with shaking (200 rpm). Samples were centrifuged at 16,000 × *g* for 5 min at room temperature, and supernatants containing the cell wall fraction were retained. Protein concentrations were determined using bicinchoninic acid (BCA) assay (Thermo Scientific, Cat. #23225) following the manufacturer's instructions. Equivalent protein concentrations of samples were diluted in SDS-PAGE loading buffer/0.1 M DTT, boiled for 5 min at 100°C, before being loaded and resolved on a SDS-PAGE gel prepared as described by previous studies (Schägger and von Jagow, 1987). Proteins were transferred to a methanol-activated PVDF membrane (Immobilon-FL, Millipore) using a wet transfer apparatus (Bio-Rad) at 100 V for 2 h. Membranes were then blocked in Odyssey blocking buffer for 1 h at room temperature. For detection of SpyCEP protein produced by GAS strains, anti-SpyCEP sera was generated by immunizing 4–6 week old BALB/c mice using purified recombinant SpyCEP (amino acids 40–683) in its inactive form (D151A S617A), and used at 1:1,000 dilution. This polyclonal antibody was cross-absorbed twice against cell-wall extract from 5448Δ*cepA* (pDCerm*cepA*) that had been impregnated onto methanol-activated PVDF membranes. Secondary antibody conjugated with a fluorescent dye (Anti-mouse IgG, Alexa Fluor® 488 Conjugate #4408, Cell Signaling Technology®) was utilized, and bound secondary antibody was detected using near-infrared (NIR) western blot detection system Odyssey CLx (Li-Cor Biosciences).

### IL-8 degradation assays

GAS strains grown to early stationary phase in THY broth were pelleted and washed twice in PBS before dilution to an OD_600_ of 0.40. GAS was then added to DMEM + 10% FCS containing 400 pg/ml of recombinant human IL-8 (BD Biosciences Pharmingen™, Cat. # 554609), and incubated at 37°C. IL-8 alone and 5448Δ*cepA* were included as negative controls.

## Ethics statement

All procedures involving animals were conducted according to Australian Code for the Care and Use of Animals for Scientific Purposes (National Health and Medical Research Council, 2013), and were approved by the University of Queensland Animal Ethics Committee (SCMB/140/16/NHMRC).

## Author contributions

AS performed experiments, analyzed the data, and drafted the paper. TB, MS, BS, LS, TR-H, VN, and MW assisted in planning of experiments, and contributed to writing the manuscript and preparation of figures. OK assisted in the bioinformatics analysis of RNAseq data. CW assisted with bioinformatics analysis and interpretation of RNAseq data. VN provided the 5448Δ*cepA* and 5448Δ*cepA* (pDCerm*cepA*) strains.

### Conflict of interest statement

The authors declare that the research was conducted in the absence of any commercial or financial relationships that could be construed as a potential conflict of interest.

## Supplementary material

Table S1 Metadata for transcriptome interaction network and pathway analysis of 5448 intracelluarly infected TEpi cells. Pathway over-representation analysis performed using InnateDB of all differentially expressed genes (adjusted *P* < 0.05, Log2FC >1 or <-1). All significantly over-represented pathways are shown (adjusted *P* < 0.05). Protein-protein interaction data type from stringDB output described.

Table S2 Metadata for transcriptome interaction network and pathway analysis of JRS4 intracellularly infected TEpi cells. Pathway over-representation analysis performed using InnateDB of all differentially expressed genes (adjusted *P* < 0.05, Log2FC >1 or <-1). All significantly over-represented pathways are shown (adjusted *P* < 0.05). Protein-protein interaction data type from stringDB output described.

Table S3 Metadata for transcriptome interaction network and pathway analysis of 5448 intracellularly infected TEpi cells in comparison to JRS4 infected cells. Pathway over-representation analysis performed using InnateDB of all differentially expressed genes (adjusted *P* < 0.05, Log2FC >1 or <-1). All significantly over-represented pathways are shown (adjusted *P* < 0.05). Protein-protein interaction data type from stringDB output described.

Figure S1 TEpi cell death during intracellular infection with JRS4 or 5448 GAS strains. Cell death measured as percentage of LDH released from TEpi cells after 6 or 24 h following GAS infection. Data are plotted as the mean ± s.e.m. and represent three independent experiments performed in triplicate and analyzed by two-way ANOVA with Tukey's post-test. Significance shown is relative to mock, unless otherwise indicated. ^*^*P* < 0.05; ^***^*P* < 0.001.

Figure S2 Invasion rate and intracellular survival of JRS4 and 5448 GAS strains during TEpi cell infection. Confluent TEpi cells were infected with either GAS strain at an MOI of 5. **(A)** Invasion rate was measured at each time post-infection by lysing TEpi cells with 0.2% Triton X-100, before performing a colony forming unit (CFU) assay. TEpi cells infected in parallel were washed and treated with gentamicin for 2 h, before being lysed and CFU assay performed. The invasion rate was measured by dividing the CFU counts of gentamicin treated TEpi cells by non-gentamicin treated wells at each time point. **(B)** Intracellular survival of GAS was measured by infecting confluent TEpi cells with either GAS strain for 2 h, before replacing the media with gentamicin-containing media for the duration of the experiment. At each time point post-infection, TEpi cells were lysed with 0.2% Triton X-100 and CFU assay performed. Results are representative of three independent experiments.

Figure S3 Amino acid sequence alignment between the *cepA* genes of 5448 and JRS4. The amino acid residues required for serine protease activity are highlighted (red boxes). An asterisk (^*^) indicates positions which have a conserved residue, a colon (:) and green lettering indicates conservative amino acid changes, and a period (.) and blue lettering indicates semi-conservative changes. Non-conservative changes are indicated by red lettering. 5448 GenBank accession number: CP008776, SpyCEP protein ID: AKK70939; JRS4 GenBank accession number: CP011414, SpyCEP protein ID: AKI75695.

Figure S4 RNAseq transcriptome network and pathway enrichment of 5448 GAS-intracellularly infected primary tonsil epithelial cells in comparison to JRS4-infected cells. **(A)** Protein-protein interaction network from the top 100 differentially expressed genes (at an adjusted *P* < 0.05) for 5448-intracellularly infected TEpi cells in comparison to JRS4-infected TEpi cells, generated using STRINGdb (*http://string-db.org/*). Network edges show the confidence of interactions, where the line thickness indicates the strength of data support. Active interaction sources include textmining, experiments, databases, co-expression, neighborhood, gene fusion and co-occurrence. Minimum required interaction score of medium confidence (0.400). Non-protein coding genes and disconnected nodes are not shown. Node color is arbitrary. **(B)** Pathway over-representation analysis of all differentially expressed genes (adjusted *P* < 0.05, Log2FC >1 or <-1) was performed using *innatedb.com*, for 5448-infected TEpi cells in comparison to JRS4-infected cells. Top 15 up-regulated pathways are shown. Green line indicates threshold for significance. Metadata from analysis results shown in Table S3.

## References

[B1] Agren K. Brauner A. Andersson J. (1998). *Haemophilus influenzae* and *Streptococcus pyogenes* group A challenge induce a Th1 type of cytokine response in cells obtained from tonsillar hypertrophy and recurrent tonsillitis. ORL J. Otorhinolaryngol. Relat. Spec. 60, 35–41. 10.1159/000027560 9519380

[B2] Andreoni F. Ogawa T. Ogawa M. Madon J. Uchiyama S. Schuepbach R. A.. (2014). The IL-8 protease SpyCEP is detrimental for Group A Streptococcus host-cells interaction and biofilm formation. Front. Microbiol. 5:339. 10.3389/fmicb.2014.00339 25071751 PMC4090674

[B3] Barnett T. C. Liebl D. Seymour L. M. Gillen C. M. Lim J. Y. Larock C. N.. (2013). The globally disseminated M1T1 clone of group A Streptococcus evades autophagy for intracellular replication. Cell Host Microbe 14, 675–682. 10.1016/j.chom.2013.11.003 24331465 PMC3918495

[B4] Bell S. Howard A. Wilson J. A. Abbot E. L. Smith W. D. Townes C. L. (2012). *Streptococcus pyogenes* infection of tonsil explants is associated with a human beta-defensin 1 response from control but not recurrent acute tonsillitis patients. Mol. Oral Microbiol. 27, 160–171. 10.1111/j.2041-1014.2012.640.x 22520386

[B5] Bestebroer J. De Haas C. J. Van Strijp J. A. (2010). How microorganisms avoid phagocyte attraction. FEMS Microbiol. Rev. 34, 395–414. 10.1111/j.1574-6976.2009.00202.x 20059549

[B6] Bisno A. L. (2001). Acute pharyngitis. N. Engl. J. Med. 344, 205–211. 10.1056/NEJM200101183440308 11172144

[B7] Buchholz K. R. Stephens R. S. (2006). Activation of the host cell proinflammatory interleukin-8 response by *Chlamydia trachomatis*. Cell. Microbiol. 8, 1768–1779. 10.1111/j.1462-5822.2006.00747.x 16803583

[B8] Breuer K. Foroushani A. K. Laird M. R. Chen C. Sribnaia A. Lo R.. (2013). InnateDB: systems biology of innate immunity and beyond-recent updates and continuing curation. Nucleic Acids Res. 41, D1228–D1233. 10.1093/nar/gks1147 23180781 PMC3531080

[B9] Chandrasekaran S. Caparon M. G. (2016). The NADase-negative variant of the *Streptococcus pyogenes* toxin NAD(+) glycohydrolase induces JNK1-mediated programmed cellular necrosis. MBio 7:e02215. 10.1128/mBio.02215-15 26838722 PMC4742715

[B10] Chatellier S. Ihendyane N. Kansal R. G. Khambaty F. Basma H. Norrby-Teglund A.. (2000). Genetic relatedness and superantigen expression in group A streptococcus serotype M1 isolates from patients with severe and nonsevere invasive diseases. Infect. Immun. 68, 3523–3534. 10.1128/IAI.68.6.3523-3534.2000 10816507 PMC97638

[B11] Courtney H. S. Ofek I. Hasty D. L. (1997). M protein mediated adhesion of M type 24 *Streptococcus pyogenes* stimulates release of interleukin-6 by HEp-2 tissue culture cells. FEMS Microbiol. Lett. 151, 65–70. 10.1111/j.1574-6968.1997.tb10395.x 9198283

[B12] Dinis M. Plainvert C. Kovarik P. Longo M. Fouet A. Poyart C. (2014). The innate immune response elicited by Group A Streptococcus is highly variable among clinical isolates and correlates with the *emm* type. PLoS ONE 9:e101464. 10.1371/journal.pone.0101464 24991887 PMC4081719

[B13] Edgar R. Domrachev M. Lash A. E. (2002). Gene Expression Omnibus: NCBI gene expression and hybridization array data repository. Nucleic Acids Res. 30, 207–210. 10.1093/nar/30.1.207 11752295 PMC99122

[B14] Edwards R. J. Taylor G. W. Ferguson M. Murray S. Rendell N. Wrigley A.. (2005). Specific C-terminal cleavage and inactivation of interleukin-8 by invasive disease isolates of *Streptococcus pyogenes*. J. Infect. Dis. 192, 783–790. 10.1086/432485 16088827

[B15] Egesten A. Eliasson M. Johansson H. M. Olin A. I. Morgelin M. Mueller A.. (2007). The CXC chemokine MIG/CXCL9 is important in innate immunity against *Streptococcus pyogenes*. J. Infect. Dis. 195, 684–693. 10.1086/510857 17262710

[B16] Eliopoulos A. G. Gallagher N. J. Blake S. M. Dawson C. W. Young L. S. (1999). Activation of the p38 mitogen-activated protein kinase pathway by Epstein-Barr virus-encoded latent membrane protein 1 coregulates interleukin-6 and interleukin-8 production. J. Biol. Chem. 274, 16085–16096. 10.1074/jbc.274.23.16085 10347160

[B17] Ellenberger T. E. Brandl C. J. Struhl K. Harrison S. C. (1992). The GCN4 basic region leucine zipper binds DNA as a dimer of uninterrupted alpha helices: crystal structure of the protein-DNA complex. Cell 71, 1223–1237. 10.1016/S0092-8674(05)80070-4 1473154

[B18] Fiebig A. Loof T. G. Babbar A. Itzek A. Koehorst J. J. Schaap P. J.. (2015). Comparative genomics of *Streptococcus pyogenes* M1 isolates differing in virulence and propensity to cause systemic infection in mice. Int. J. Med. Microbiol. 305, 532–543. 10.1016/j.ijmm.2015.06.002 26129624

[B19] Gerber M. A. Baltimore R. S. Eaton C. B. Gewitz M. Rowley A. H. Shulman S. T.. (2009). Prevention of rheumatic fever and diagnosis and treatment of acute *Streptococcal pharyngitis*: a scientific statement from the American Heart Association Rheumatic Fever, Endocarditis, and Kawasaki Disease Committee of the Council on Cardiovascular Disease in the Young, the Interdisciplinary Council on Functional Genomics and Translational Biology, and the Interdisciplinary Council on Quality of Care and Outcomes Research: endorsed by the American Academy of Pediatrics. Circulation 119, 1541–1551. 10.1161/CIRCULATIONAHA.109.191959 19246689

[B20] Gudjonsson J. E. Thorarinsson A. M. Sigurgeirsson B. Kristinsson K. G. Valdimarsson H. (2003). Streptococcal throat infections and exacerbation of chronic plaque psoriasis: a prospective study. Br. J. Dermatol. 149, 530–534. 10.1046/j.1365-2133.2003.05552.x 14510985

[B21] Hai T. Curran T. (1991). Cross-family dimerization of transcription factors Fos/Jun and ATF/CREB alters DNA binding specificity. Proc. Natl. Acad. Sci. U.S.A. 88, 3720–3724. 10.1073/pnas.88.9.3720 1827203 PMC51524

[B22] Heinz S. Benner C. Spann N. Bertolino E. Lin Y. C. Laslo P.. (2010). Simple combinations of lineage-determining transcription factors prime cis-regulatory elements required for macrophage and B cell identities. Mol. Cell 38, 576–589. 10.1016/j.molcel.2010.05.004 20513432 PMC2898526

[B23] Hidalgo-Grass C. Mishalian I. Dan-Goor M. Belotserkovsky I. Eran Y. Nizet V.. (2006). A streptococcal protease that degrades CXC chemokines and impairs bacterial clearance from infected tissues. EMBO J. 25, 4628–4637. 10.1038/sj.emboj.7601327 16977314 PMC1589981

[B24] Jundi K. Greene C. M. (2015). Transcription of interleukin-8: how altered regulation can affect cystic fibrosis lung disease. Biomolecules 5, 1386–1398. 10.3390/biom5031386 26140537 PMC4598756

[B25] Kaunisto A. Henry W. S. Montaser-Kouhsari L. Jaminet S. C. Oh E. Y. Zhao L.. (2015). NFAT1 promotes intratumoral neutrophil infiltration by regulating IL8 expression in breast cancer. Mol. Oncol. 9, 1140–1154. 10.1016/j.molonc.2015.02.004 25735562 PMC4439371

[B26] Klenk M. Koczan D. Guthke R. Nakata M. Thiesen H. J. Podbielski A.. (2005). Global epithelial cell transcriptional responses reveal *Streptococcus pyogenes* Fas regulator activity association with bacterial aggressiveness. Cell. Microbiol. 7, 1237–1250. 10.1111/j.1462-5822.2005.00548.x 16098212

[B27] Klenk M. Nakata M. Podbielski A. Skupin B. Schroten H. Kreikemeyer B. (2007). *Streptococcus pyogenes* serotype-dependent and independent changes in infected HEp-2 epithelial cells. ISME J. 1, 678–692. 10.1038/ismej.2007.54 18059492

[B28] Liao Y. Smyth G. K. Shi W. (2013). The Subread aligner: fast, accurate and scalable read mapping by seed-and-vote. Nucleic Acids Res. 41:e108. 10.1093/nar/gkt214 23558742 PMC3664803

[B29] Linge H. M. Sastalla I. Nitsche-Schmitz D. P. Egesten A. Frick I. M. (2007). Protein FOG is a moderate inducer of MIG/CXCL9, and group G streptococci are more tolerant than group A streptococci to this chemokine's antibacterial effect. Microbiology 153, 3800–3808. 10.1099/mic.0.2007/009647-0 17975089

[B30] Lodowski D. T. Palczewski K. (2009). Chemokine receptors and other G protein-coupled receptors. Curr. Opin. 4, 88–95. 10.1097/COH.0b013e3283223d8d 19339946 PMC2771364

[B31] Medina E. Anders D. Chhatwal G. S. (2002). Induction of NF-kappaB nuclear translocation in human respiratory epithelial cells by group A streptococci. Microb. Pathog. 33, 307–313. 10.1006/mpat.2002.0532 12495677

[B32] Murphy C. McGurk M. Pettigrew J. Santinelli A. Mazzucchelli R. Johnston P. G.. (2005). Nonapical and cytoplasmic expression of interleukin-8, CXCR1, and CXCR2 correlates with cell proliferation and microvessel density in prostate cancer. Clin. Cancer Res. 11, 4117–4127. 10.1158/1078-0432.CCR-04-1518 15930347

[B33] Nakagawa I. Nakata M. Kawabata S. Hamada S. (2004). Transcriptome analysis and gene expression profiles of early apoptosis-related genes in *Streptococcus pyogenes*-infected epithelial cells. Cell. Microbiol. 6, 939–952. 10.1111/j.1462-5822.2004.00412.x 15339269

[B34] National Health and Medical Research Council, (2013). Australian Code for the Care and Use of Animals for Scientific Purposes, 8th Edn. Canberra, ACT: National Health and Medical Research Council.

[B35] Norgren M. Caparon M. G. Scott J. R. (1989). A method for allelic replacement that uses the conjugative transposon Tn916: deletion of the emm6.1 allele in *Streptococcus pyogenes* JRS4. Infect. Immun. 57, 3846–3850. 2553613 10.1128/iai.57.12.3846-3850.1989 PMC259915

[B36] Oeckinghaus A. Ghosh S. (2009). The NF-kappaB family of transcription factors and its regulation. Cold Spring Harb. Perspect. Biol. 1:a000034. 10.1101/cshperspect.a000034 20066092 PMC2773619

[B37] Oshlack A. Robinson M. D. Young M. D. (2010). From RNA-seq reads to differential expression results. Genome Biol. 11:220. 10.1186/gb-2010-11-12-220 21176179 PMC3046478

[B38] Osterlund A. Popa R. Nikkilä T. Scheynius A. Engstrand L. (1997). Intracellular reservoir of *Streptococcus pyogenes in vivo*: a possible explanation for recurrent pharyngotonsillitis. Laryngoscope 107, 640–647. 10.1097/00005537-199705000-00016 9149167

[B39] Persson S. T. Wilk L. Mörgelin M. Herwald H. (2015). Vigilant keratinocytes trigger pathogen-associated molecular pattern signaling in response to streptococcal M1 protein. Infect. Immun. 83, 4673–4681. 10.1128/IAI.00887-15 26416902 PMC4645376

[B40] Port G. C. Paluscio E. Caparon M. G. (2015). Complete genome sequences of *emm6 Streptococcus pyogenes* JRS4 and parental strain D471. Genome Announc. 3:e00725–15. 10.1128/genomeA.00725-15 26139722 PMC4490850

[B41] Ralph A. P. Carapetis J. R. (2013). Group A streptococcal diseases and their global burden. Curr. Top. Microbiol. Immunol. 368, 1–27. 10.1007/82_2012_280 23242849

[B42] Rao A. Luo C. Hogan P. G. (1997). Transcription factors of the NFAT family: regulation and function. Annu. Rev. Immunol. 15, 707–747. 10.1146/annurev.immunol.15.1.707 9143705

[B43] Reichle A. (2010). From Molecular to Modular Tumor Therapy : Tumors Are Reconstructible Communicatively Evolving Systems. New York, NY; Dordrecht: Springer.

[B44] Ritchie M. E. Phipson B. Wu D. Hu Y. Law C. W. Shi W.. (2015). *limma* powers differential expression analyses for RNA-sequencing and microarray studies. Nucleic Acids Res. 43:e47. 10.1093/nar/gkv007 25605792 PMC4402510

[B45] Rizzo A. Losacco A. Carratelli C. R. Domenico M. D. Bevilacqua N. (2013). *Lactobacillus plantarum* reduces *Streptococcus pyogenes* virulence by modulating the IL-17, IL-23 and Toll-like receptor 2/4 expressions in human epithelial cells. Int. Immunopharmacol. 17, 453–461. 10.1016/j.intimp.2013.07.005 23892030

[B46] Roberts A. L. Connolly K. L. Kirse D. J. Evans A. K. Poehling K. A. Peters T. R.. (2012). Detection of group A Streptococcus in tonsils from pediatric patients reveals high rate of asymptomatic streptococcal carriage. BMC Pediatr. 12:3. 10.1186/1471-2431-12-3 22230361 PMC3279307

[B47] Robinson M. D. Oshlack A. (2010). A scaling normalization method for differential expression analysis of RNA-seq data. Genome Biol. 11:R25. 10.1186/gb-2010-11-3-r25 20196867 PMC2864565

[B48] Rodriguez-Iturbe B. Musser J. M. (2008). The current state of poststreptococcal glomerulonephritis. J. Am. Soc. Nephrol. 19, 1855–1864. 10.1681/ASN.2008010092 18667731

[B49] Schägger H. von Jagow G. (1987). Tricine-sodium dodecyl sulfate-polyacrylamide gel electrophoresis for the separation of proteins in the range from 1 to 100 kDa. Anal. Biochem. 166, 368–379. 10.1016/0003-2697(87)90587-2 2449095

[B50] Soderholm A. T. Barnett T. C. Sweet M. J. Walker M. J. (2017). Group A streptococcal pharyngitis: immune responses involved in bacterial clearance and GAS-associated immunopathologies. J. Leukoc. Biol. 103, 193–213. 10.1189/jlb.4MR0617-227RR 28951419

[B51] Spencer J. A. Misra R. P. (1996). Expression of the serum response factor gene is regulated by serum response factor binding sites. J. Biol. Chem. 271, 16535–16543. 10.1074/jbc.271.28.16535 8663310

[B52] Stanley A. C. Lacy P. (2010). Pathways for cytokine secretion. Physiology 25, 218–229. 10.1152/physiol.00017.2010 20699468

[B53] Sumby P. Zhang S. Whitney A. R. Falugi F. Grandi G. Graviss E. A.. (2008). A chemokine-degrading extracellular protease made by group A Streptococcus alters pathogenesis by enhancing evasion of the innate immune response. Infect. Immun. 76, 978–985. 10.1128/IAI.01354-07 18174342 PMC2258835

[B54] Sun L. Ye R. D. (2012). Role of G protein-coupled receptors in inflammation. Acta Pharmacol. Sin. 33, 342–350. 10.1038/aps.2011.200 22367283 PMC4085652

[B55] Szklarczyk D. Morris J. H. Cook H. Kuhn M. Wyder S. Simonovic M.. (2017). The STRING database in 2017: quality-controlled protein-protein association networks, made broadly accessible. Nucleic Acids Res. 45, D362–D368. 10.1093/nar/gkw937 27924014 PMC5210637

[B56] Tsai P. J. Chen Y. H. Hsueh C. H. Hsieh H. C. Liu Y. H. Wu J. J.. (2006). *Streptococcus pyogenes* induces epithelial inflammatory responses through NF-kappaB/MAPK signaling pathways. Microbes Infect. 8, 1440–1449. 10.1016/j.micinf.2006.01.002 16702013

[B57] Turner C. E. Kurupati P. Jones M. D. Edwards R. J. Sriskandan S. (2009). Emerging role of the interleukin-8 cleaving enzyme SpyCEP in clinical *Streptococcus pyogenes* infection. J. Infect. Dis. 200, 555–563. 10.1086/603541 19591574 PMC2820315

[B58] Ulukus M. Ulukus E. C. Tavmergen Goker E. N. Tavmergen E. Zheng W. Arici A. (2009). Expression of interleukin-8 and monocyte chemotactic protein 1 in women with endometriosis. Fertil. Steril. 91, 687–693. 10.1016/j.fertnstert.2007.12.067 18314120

[B59] Viciani E. Montagnani F. Tavarini S. Tordini G. Maccari S. Morandi M.. (2016). Paediatric obstructive sleep apnoea syndrome (OSAS) is associated with tonsil colonisation by *Streptococcus pyogenes*. Sci. Rep. 6:20609. 10.1038/srep20609 26860261 PMC4748291

[B60] Walker M. J. Barnett T. C. McArthur J. D. Cole J. N. Gillen C. M. Henningham A.. (2014). Disease manifestations and pathogenic mechanisms of Group A Streptococcus. Clin. Microbiol. Rev. 27, 264–301. 10.1128/CMR.00101-13 24696436 PMC3993104

[B61] Wang B. Dileepan T. Briscoe S. Hyland K. A. Kang J. Khoruts A.. (2010). Induction of TGF-beta1 and TGF-beta1-dependent predominant Th17 differentiation by group A streptococcal infection. Proc. Natl. Acad. Sci. U.S.A. 107, 5937–5942. 10.1073/pnas.0904831107 20231435 PMC2851870

[B62] Wang B. Ruiz N. Pentland A. Caparon M. (1997). Keratinocyte proinflammatory responses to adherent and nonadherent group A streptococci. Infect. Immun. 65, 2119–2126. 9169741 10.1128/iai.65.6.2119-2126.1997 PMC175293

[B63] Wells C. A. Mosbergen R. Korn O. Choi J. Seidenman N. Matigian N. A.. (2013). Stemformatics: visualisation and sharing of stem cell gene expression. Stem Cell Res. 10, 387–395. 10.1016/j.scr.2012.12.003 23466562

[B64] Wessels M. R. (2016). Pharyngitis and scarlet fever, in Streptococcus pyogenes: Basic Biology to Clinical Manifestations [Internet], eds Ferretti J. J. Stevens D. L. Fischetti V. A. (Oklahoma City, OK: University of Oklahoma Health Sciences Center). 26866208

[B65] Ye R. D. (2001). Regulation of nuclear factor kappaB activation by G-protein-coupled receptors. J. Leukoc. Biol. 70, 839–848. 11739545

[B66] Zingaretti C. Falugi F. Nardi-Dei V. Pietrocola G. Mariani M. Liberatori S.. (2010). *Streptococcus pyogenes* SpyCEP: a chemokine-inactivating protease with unique structural and biochemical features. FASEB J. 24, 2839–2848. 10.1096/fj.09-145631 20339024

[B67] Zinkernagel A. S. Timmer A. M. Pence M. A. Locke J. B. Buchanan J. T. Turner C. E.. (2008). The IL-8 protease SpyCEP/ScpC of group A Streptococcus promotes resistance to neutrophil killing. Cell Host Microbe 4, 170–178. 10.1016/j.chom.2008.07.002 18692776 PMC2631432

