# Supplementary material for: Group A Streptococcus M1T1 Intracellular Infection of Primary Tonsil Epithelial Cells Dampens Levels of Secreted IL-8 Through the Action of SpyCEP
Source: Front Cell Infect Microbiol. 2018 May 17;8:160. doi: 10.3389/fcimb.2018.00160 (PMC5966554; doi:10.3389/fcimb.2018.00160)
Supplement: Table S1 — Metadata for transcriptome interaction network and pathway analysis of 5448 intracelluarly infected TEpi cells. Pathway over-representation analysis performed using InnateDB of all differentially expressed genes (adjusted P < 0.05, Log2FC >1 or <-1). All significantly over-represented pathways are shown (adjusted P < 0.05). Protein-protein interaction data type from stringDB output described. [file Table_1.pdf]

Table S1. Metadata for transcriptome interaction network and pathway analysis of 5448 intracelluarly infected TEpi cells in comparison to mock TEpi cells.

Gene symbol	Full name	Log2FC gene expression in comparison to Mock cells	Adjusted P-value	Protein-protein interaction data type from Stringdb.com	Upregulated pathways from Innatedb.com
FOSB	FBJ murine osteosarcoma viral oncogene homolog B	5.412	7.89E-06	experimentally determined, textmining	AP-1 transcription factor network, Osteoclast differentiation
TNF	tumor necrosis factor	5.228	7.66E-06	coexpression, textmining	Calcineurin-regulated NFAT-dependent transcription in lymphocytes, Cytokine-cytokine receptor interaction, Osteoclast differentiation, GPCR signaling, IL23-mediated signaling events, MAPK signaling pathway, Signal transduction through il1r, NOD-like receptor signaling pathway, TWEAK, amb2 Integrin signaling, Downstream signaling in naïve CD8+ T cells, Toll-like receptor signaling pathway, Hematopoietic cell lineage, Hepatitis C, Keratinocyte differentiation, Leishmaniasis, HIV-1 Nef: Negative effector of Fas and TNF-alpha, T cell receptor signaling pathway, TNFR1 signaling pathway, TNF receptor signaling pathway
CXCL2	chemokine (C-X-C motif) ligand 2	4.717	3.18E-05	coexpression, curated databases, protein homology, textmining	Cytokine-cytokine receptor interaction, GPCR ligand binding, Class A/1 (Rhodopsin-like receptors), NOD-like receptor signaling pathway, Chemokine receptors bind chemokines, Signaling by GPCR, Peptide ligand-binding receptors, GPCR downstream signaling, G alpha (i) signalling events
IL8	interleukin 8	4.657	2.17E-05	coexpression, curated databases, protein homology, textmining	AP-1 transcription factor network, ATF-2 transcription factor network, Calcineurin-regulated NFAT-dependent transcription in lymphocytes, Cytokine-cytokine receptor interaction, Validated transcriptional targets of AP1 family members Fra1 and Fra2, GPCR signaling, GPCR ligand binding, Class A/1 (Rhodopsin-like receptors), NOD-like receptor signaling pathway, Chemokine receptors bind chemokines, Glucocorticoid receptor regulatory network, Signaling by GPCR, Toll-like receptor signaling pathway, Senescence-Associated Secretory Phenotype (SASP), Peptide ligand-binding receptors, Hepatitis C, GPCR downstream signaling, G alpha (i) signalling events
ATF3	activating transcription factor 3	4.384	7.98E-05	experimentally determined, coexpression, protein homology, textmining	AP-1 transcription factor network, ATF-2 transcription factor network, Direct p53 effectors
EGR2	early growth response 2	4.337	1.07E-05	experimentally determined, coexpression, textmining	Calcineurin-regulated NFAT-dependent transcription in lymphocytes

PPP1R15A	protein phosphatase 1, regulatory subunit 15A	4.057	6.32E-07	coexpression, textmining	None
EGR4	early growth response 4	3.975	1.33E-04	experimentally determined, textmining	Calcineurin-regulated NFAT-dependent transcription in lymphocytes, Downstream signaling in naïve CD8+ T cells
TNFAIP3	tumor necrosis factor, alpha-induced protein 3	3.969	9.07E-06	experimentally determined, coexpression, textmining	CD40/CD40L signaling, NOD-like receptor signaling pathway, Tnfr2 signaling pathway, RIG-I/MDA5 mediated induction of IFN-alpha/beta pathways, TNF receptor signaling pathway
TRAF1	TNF receptor-associated factor 1	3.928	6.14E-05	experimentally determined, curated databases, coexpression, textmining	CD40/CD40L signaling, TWEAK, Tnfr2 signaling pathway, RANKL, Keratinocyte differentiation, HIV-1 Nef: Negative effector of Fas and TNF-alpha, TNF receptor signaling pathway
CCL20	chemokine (C-C motif) ligand 20	3.897	2.41E-05	coexpression, curated databases, textmining	Cytokine-cytokine receptor interaction, GPCR ligand binding, Class A/1 (Rhodopsin-like receptors), NOD-like receptor signaling pathway, Chemokine receptors bind chemokines, Signaling by GPCR, Peptide ligand-binding receptors, GPCR downstream signaling, G
LIF	leukemia inhibitory factor	3.869	1.34E-04	curated databases, textmining	Cytokine-cytokine receptor interaction, Validated transcriptional targets of AP1 family members Fra1 and Fra2, GPCR signaling, Direct p53 effectors, Jak-STAT signaling pathway
HBEGF	heparin-binding EGF-like growth factor	3.869	4.81E-06	curated databases, textmining	GPCR signaling, Signaling by GPCR, ErbB receptor signaling network
IRF1	interferon regulatory factor 1	3.811	7.98E-05	experimentally determined, coexpression, curated databases, textmining	Glucocorticoid receptor regulatory network, Cytokine Signaling in Immune system, IL6-mediated signaling events, Interferon alpha/beta signaling, Hepatitis C, RIG-I/MDA5 mediated induction of IFN-alpha/beta pathways
EGR1	early growth response 1	3.811	1.23E-03	experimentally determined, coexpression, curated databases, textmining	AP-1 transcription factor network, Calcineurin-regulated NFAT-dependent transcription in lymphocytes, Glucocorticoid receptor regulatory network, Cytokine Signaling in Immune system, Downstream signaling in naïve CD8+ T cells, Interferon alpha/beta signaling, Oncostatin_M

IL6	interleukin 6 (interferon, beta 2)	3.739	5.25E-04	coexpression, curated databases, textmining	AP-1 transcription factor network, ATF-2 transcription factor network, Cytokine-cytokine receptor interaction, Validated transcriptional targets of AP1 family members Fra1 and Fra2, GPCR signaling, IL23-mediated signaling events, Signal transduction through il1r, NOD-like receptor signaling pathway, Activated TLR4 signalling, Toll Like Receptor 4 (TLR4) Cascade, Toll-Like Receptors Cascades, Jak-STAT signaling pathway, MyD88 dependent cascade initiated on endosome, Toll Like Receptor 7/8 (TLR7/8) Cascade, Glucocorticoid receptor regulatory network, Cytokine Signaling in Immune system, Signaling by GPCR, amb2 Integrin signaling, Signaling by Interleukins, Toll Like Receptor 9 (TLR9) Cascade, MyD88:Mal cascade initiated on plasma membrane, Toll Like Receptor 2 (TLR2) Cascade, Toll Like Receptor TLR1:TLR2 Cascade, Toll Like Receptor TLR6:TLR2 Cascade, IL6-mediated signaling events, Toll-like receptor signaling pathway, MyD88 cascade initiated on plasma membrane, TRAF6 mediated induction of NFkB and MAP kinases upon TLR7/8 or 9 activation, Toll Like Receptor 10 (TLR10) Cascade, Toll Like Receptor 5 (TLR5) Cascade, Hematopoietic cell lineage, MyD88-independent cascade, TRIF-mediated TLR3/TLR4 signaling, Toll Like Receptor 3 (TLR3) Cascade, Senescence-Associated Secretory Phenotype (SASP)
BIRC3	baculoviral IAP repeat containing 3	3.688	3.20E-05	experimentally determined, coexpression, curated databases, textmining	CD40/CD40L signaling, NOD-like receptor signaling pathway, Activated TLR4 signalling, Toll Like Receptor 4 (TLR4) Cascade, Toll-Like Receptors Cascades, MyD88-independent cascade, TRIF-mediated TLR3/TLR4 signaling, Toll Like Receptor 3 (TLR3) Cascade, Keratinocyte differentiation, HIV-1 Nef: Negative effector of Fas and TNF-alpha, TNF receptor signaling pathway
NR4A1	nuclear receptor subfamily 4, group A, member 1	3.674	1.58E-04	experimentally determined, coexpression, textmining	MAPK signaling pathway, Glucocorticoid receptor regulatory network
CD83	CD83 molecule	3.665	6.14E-05	coexpression, textmining	None
MAFF	v-maf musculoaponeurotic fibrosarcoma oncogene homolog F (avian)	3.605	9.07E-06	experimentally determined, coexpression, textmining	Oxidative stress induced gene expression via nrf2
PTGS2	prostaglandin-endoperoxide synthase 2 (prostaglandin G/H synthase and cyclooxygenase)	3.471	4.77E-04	coexpression, textmining	Calcineurin-regulated NFAT-dependent transcription in lymphocytes, Calcium signaling in the CD4+ TCR pathway, Leishmaniasis

NR4A3	nuclear receptor subfamily 4, group A, member 3	3.401	5.08E-04	experimentally determined, textmining	None
EGR3	early growth response 3	3.281	9.07E-06	experimentally determined, coexpression, textmining	Calcineurin-regulated NFAT-dependent transcription in lymphocytes
GADD45B	growth arrest and DNA-damage-inducible, beta	3.279	1.76E-05	experimentally determined, protein homology, textmining	MAPK signaling pathway, IL12-mediated signaling events
SOCS3	suppressor of cytokine signaling 3	3.276	9.07E-06	coexpression, curated databases, textmining	ATF-2 transcription factor network, Osteoclast differentiation, IL23-mediated signaling events, Jak-STAT signaling pathway, Cytokine Signaling in Immune system, Signaling by Interleukins, IL6-mediated signaling events, Interferon alpha/beta signaling, Hepatitis C, Oncostatin_M
DUSP5	dual specificity phosphatase 5	3.143	1.46E-05	experimentally determined, coexpression, textmining	ATF-2 transcription factor network, MAPK signaling pathway, Direct p53 effectors
CSF2	colony stimulating factor 2 (granulocyte-macrophage)	2.968	6.10E-04	coexpression, textmining	AP-1 transcription factor network, Calcineurin-regulated NFAT-dependent transcription in lymphocytes, Cytokine-cytokine receptor interaction, GPCR signaling, Calcium signaling in the CD4+ TCR pathway, Jak-STAT signaling pathway, Glucocorticoid receptor regulatory network, Cytokine Signaling in Immune system, Signaling by GPCR, Signaling by Interleukins, Hematopoietic cell lineage, GPCR downstream signaling, T cell receptor signaling pathway
NFKBIE	nuclear factor of kappa light polypeptide gene enhancer in B-cells inhibitor, epsilon	2.949	1.07E-05	experimentally determined, coexpression, textmining	T cell receptor signaling pathway, TNFR1 signaling pathway, B cell receptor signaling pathway
FOSL1	FOS-like antigen 1	2.891	3.82E-06	experimentally determined, coexpression, protein homology, curated databases, textmining	AP-1 transcription factor network, Calcineurin-regulated NFAT-dependent transcription in lymphocytes, Osteoclast differentiation, Validated transcriptional targets of AP1 family members Fra1 and Fra2, Calcium signaling in the CD4+ TCR pathway, Downstream signaling in naïve CD8+ T cells,
PMAIP1	phorbol-12-myristate-13-acetate-induced protein 1	2.875	5.28E-06	experimentally determined, curated databases, textmining	Direct p53 effectors
EDN1	endothelin 1	2.833	7.07E-05	textmining	AP-1 transcription factor network, GPCR signaling, GPCR ligand binding, Class A/1 (Rhodopsin-like receptors), Signaling by GPCR, Peptide ligand-binding receptors, GPCR downstream signaling

BCL2A1	BCL2-related protein A1	2.825	5.55E-05	experimentally determined, curated databases, textmining	BCR signaling pathway
CXCL3	chemokine (C-X-C motif) ligand 3	2.785	8.39E-05	coexpression, curated databases, protein homology, textmining	Cytokine-cytokine receptor interaction, GPCR ligand binding, Class A/1 (Rhodopsin-like receptors), Chemokine receptors bind chemokines, Signaling by GPCR, Peptide ligand-binding receptors, GPCR downstream signaling, G alpha (i) signalling events
BBC3	BCL2 binding component 3	2.710	3.30E-04	experimentally determined, curated databases, textmining	Direct p53 effectors
SERPINB2	serpin peptidase inhibitor, clade B (ovalbumin), member 2	2.582	2.35E-04	curated databases, textmining	None
PLAUR	plasminogen activator, urokinase receptor	2.572	2.95E-06	coexpression, curated databases, textmining	Validated transcriptional targets of AP1 family members Fra1 and Fra2, amb2 Integrin signaling
ZFP36	zinc finger protein 36, C3H type, homolog (mouse)	2.524	2.25E-05	experimentally determined, coexpression, textmining	None
PRDM1	PR domain containing 1, with ZNF domain	2.449	1.67E-05	experimentally determined, textmining	Direct p53 effectors
PIM1	pim-1 oncogene	2.446	1.50E-04	experimentally determined, curated databases, textmining	Jak-STAT signaling pathway
PTX3	pentraxin 3, long	3.721	6.06E-04	coexpression, textmining	None
IL1RL1	interleukin 1 receptor-like 1	3.396	5.31E-04	textmining	None
IL24	interleukin 24	2.485	1.51E-04	textmining	Cytokine-cytokine receptor interaction, IL23-mediated signaling events
