# Supplementary material for: Group A Streptococcus M1T1 Intracellular Infection of Primary Tonsil Epithelial Cells Dampens Levels of Secreted IL-8 Through the Action of SpyCEP
Source: Front Cell Infect Microbiol. 2018 May 17;8:160. doi: 10.3389/fcimb.2018.00160 (PMC5966554; doi:10.3389/fcimb.2018.00160)
Supplement: Table S2 — Metadata for transcriptome interaction network and pathway analysis of JRS4 intracellularly infected TEpi cells. Pathway over-representation analysis performed using InnateDB of all differentially expressed genes (adjusted P < 0.05, Log2FC >1 or <-1). All significantly over-represented pathways are shown (adjusted P < 0.05). Protein-protein interaction data type from stringDB output described. [file Table_2.pdf]

Table S2. Metadata for transcriptome interaction network and pathway analysis of JRS4 intracellularly infected TEpi cells in comparison to mock TEpi cells.

Gene symbol	Full name	Log2FC gene expression in comparison to Mock cells	Adjusted P-value	Protein-protein interaction data type from Stringdb.com	Upregulated pathways from Innatedb.com
ATF3	activating transcription factor 3	3.299	3.59E-03	experimentally determined, co-expression, protein homology, curated databases, textmining	AP-1 transcription factor network, ATF-2 transcription factor network, Direct p53 effectors
PTGS2	prostaglandin-endoperoxide synthase 2 (prostaglandin G/H synthase and cyclooxygenase)	3.207	6.56E-03	curated databases, textmining	Calcineurin-regulated NFAT-dependent transcription in lymphocytes, Calcium signaling in the CD4+ TCR pathway, Leishmaniasis
IL8	interleukin 8	3.063	2.46E-03	co-expression, protein homology, curated databases, textmining	AP-1 transcription factor network, Cytokine-cytokine receptor interaction, ATF-2 transcription factor network, Calcineurin-regulated NFAT-dependent transcription in lymphocytes, GPCR signaling, Chemokine receptors bind chemokines, GPCR ligand binding, Signaling by GPCR, Class A/1 (Rhodopsin-like receptors), NOD-like receptor signaling pathway, Validated transcriptional targets of AP1 family members Fra1 and Fra2, Peptide ligand-binding receptors, Amoebiasis, GPCR downstream signaling, G alpha (i) signalling events, Nfkb activation by nontypeable hemophilus influenzae, Malaria
IL36G	interleukin 36, gamma	3.034	8.88E-05	co-expression, textmining	None
CXCL2	chemokine (C-X-C motif) ligand 2	2.897	4.46E-03	co-expression, protein homology, curated databases, textmining	Cytokine-cytokine receptor interaction, Chemokine receptors bind chemokines, GPCR ligand binding, Signaling by GPCR, Class A/1 (Rhodopsin-like receptors), NOD-like receptor signaling pathway, Peptide ligand-binding receptors, GPCR downstream signaling, G alpha (i) signalling events
IL6	interleukin 6 (interferon, beta 2)	2.847	1.82E-02	co-expresssion, curated databases, textmining	AP-1 transcription factor network, Cytokine-cytokine receptor interaction, ATF-2 transcription factor network, GPCR signaling, IL23-mediated signaling events, Signaling by GPCR, NOD-like receptor signaling pathway, Validated transcriptional targets of AP1 family members Fra1 and Fra2, Amoebiasis, Jak-STAT signaling pathway, Hematopoietic cell lineage, Signal transduction through il1r, Graft-versus-host disease, Malaria, Il-10 anti-inflammatory signaling pathway

CLDN4	claudin 4	2.743	5.47E-04	curated databases, protein homology, co-expression, textmining	None
TNF	tumor necrosis factor	2.606	3.08E-03	co-expression, textmining	Cytokine-cytokine receptor interaction, Calcineurin-regulated NFAT-dependent transcription in lymphocytes, GPCR signaling, IL23-mediated signaling events, NOD-like receptor signaling pathway, Amoebiasis, Hematopoietic cell lineage, Signal transduction through il1r, Graft-versus-host disease, Osteoclast differentiation, Nfkb activation by nontypeable hemophilus influenzae, Malaria, Il-10 anti-inflammatory signaling pathway, Leishmaniasis
SERPINB2	serpin peptidase inhibitor, clade B (ovalbumin), member 2	2.446	2.86E-03	curated databases, textmining	Amoebiasis
MMP3	matrix metallopeptidase 3 (stromelysin 1, progelatinase)	2.337	2.41E-02	textmining	Signaling by GPCR
CXCL3	chemokine (C-X-C motif) ligand 3	2.288	2.63E-03	co-expression, protein homology, curated databases, textmining	Cytokine-cytokine receptor interaction, Chemokine receptors bind chemokines, GPCR ligand binding, Signaling by GPCR, Class A/1 (Rhodopsin-like receptors), Peptide ligand-binding receptors, GPCR downstream signaling, G alpha (i) signalling events
IL1RL1	interleukin 1 receptor-like 1	2.282	3.35E-02	experimentally determined, textmining	None
TNFAIP3	tumor necrosis factor, alpha-induced protein 3	2.271	2.00E-03	experimentally determined, co-expression, textmining	NOD-like receptor signaling pathway
DUSP1	dual specificity phosphatase 1	2.141	1.48E-03	experimentally determined, co-expression, curated databases, textmining	AP-1 transcription factor network, ATF-2 transcription factor network, Direct p53 effectors, Nfkb activation by nontypeable hemophilus influenzae
DLX2	distal-less homeobox 2	2.136	2.63E-02	textmining	None
IL24	interleukin 24	2.006	4.50E-03	co-expression, textmining	Cytokine-cytokine receptor interaction, IL23-mediated signaling events, Jak-STAT signaling pathway
OCLN	occludin	2.004	2.86E-03	experimentally determined, textmining	None
PIM1	pim-1 oncogene	1.931	5.21E-03	experimentally determined, curated databases, textmining	Jak-STAT signaling pathway

EGR2	early growth response 2	1.930	1.08E-02	experimentally determined, textmining	Calcineurin-regulated NFAT-dependent transcription in lymphocytes
FOSB	FBJ murine osteosarcoma viral oncogene homolog B	1.929	2.03E-02	experimentally determined, curated databases, textmining	AP-1 transcription factor network, Osteoclast differentiation
PPP1R15A	protein phosphatase 1, regulatory subunit 15A	1.921	3.46E-04	co-expresssion, curated databases, textmining	None
DUSP5	dual specificity phosphatase 5	1.909	2.62E-03	experimentally determined, co-expression, textmining	ATF-2 transcription factor network, Direct p53 effectors
SOCS3	suppressor of cytokine signaling 3	1.830	2.62E-03	co-expresssion, curated databases, textmining	ATF-2 transcription factor network, IL23-mediated signaling events, Jak-STAT signaling pathway, Osteoclast differentiation
PRDM1	PR domain containing 1, with ZNF domain	1.829	1.48E-03	textmining	Direct p53 effectors
HBEGF	heparin-binding EGF-like growth factor	1.825	2.94E-03	curated databases, textmining	GPCR signaling, Signaling by GPCR, ErbB receptor signaling network, ErbB signaling pathway
PDE4B	phosphodiesterase 4B, cAMP-specific	1.810	7.73E-03	experimentally determined, textmining	Signaling by GPCR, GPCR downstream signaling
PLAUR	plasminogen activator, urokinase receptor	1.771	2.63E-04	co-expresssion, curated databases, textmining	Validated transcriptional targets of AP1 family members Fra1 and Fra2
CD274	CD274 molecule	1.768	4.26E-04	co-expression, textmining	None
ZFP36	zinc finger protein 36, C3H type, homolog (mouse)	1.745	2.00E-03	co-expresssion, curated databases, textmining	None
HCAR2	hydroxycarboxylic acid receptor 2	1.727	1.48E-03	curated databases, textmining	GPCR ligand binding, Signaling by GPCR, Class A/1 (Rhodopsin-like receptors), GPCR downstream signaling, G alpha (i) signalling events
PMAIP1	phorbol-12-myristate-13-acetate-induced protein 1	1.695	1.48E-03	textmining	Direct p53 effectors
CLDN1	Rho family GTPase 1	1.691	1.10E-02	textmining	None
RND1	Rho family GTPase 1	1.691	1.10E-02	experimentally determined, textmining	None
LMO7	LIM domain 7	1.665	3.59E-03	curated databases, textmining	None

FOXA1	forkhead box A1	1.645	5.75E-03	textmining	Direct p53 effectors
RGS2	regulator of G-protein signaling 2, 24kDa	1.637	3.25E-02	curated databases, textmining	GPCR signaling, Signaling by GPCR, GPCR downstream signaling, G alpha (i) signalling events
CCL20	chemokine (C-C motif) ligand 20	1.589	2.82E-02	co-expresssion, curated databases, textmining	Cytokine-cytokine receptor interaction, Chemokine receptors bind chemokines, GPCR ligand binding, Signaling by GPCR, Class A/1 (Rhodopsin-like receptors), Peptide ligand-binding receptors, GPCR downstream signaling, G alpha (i) signalling events
CXCL1	chemokine (C-X-C motif) ligand 1 (melanoma growth stimulating activity, alpha)	1.524	1.09E-02	co-expression, protein homology, curated databases, textmining	Cytokine-cytokine receptor interaction, IL23-mediated signaling events, Chemokine receptors bind chemokines, GPCR ligand binding, Signaling by GPCR, Class A/1 (Rhodopsin-like receptors), NOD-like receptor signaling pathway, Peptide ligand-binding receptors, Amoebiasis, GPCR downstream signaling, G alpha (i) signalling events
FOSL1	FOS-like antigen 1	1.517	1.48E-03	experimentally determined, co-expression, protein homology, curated databases, textmining	AP-1 transcription factor network, Calcineurin-regulated NFAT-dependent transcription in lymphocytes, Validated transcriptional targets of AP1 family members Fra1 and Fra2, Calcium signaling in the CD4+ TCR pathway, Osteoclast differentiation
MMP10	matrix metallopeptidase 10 (stromelysin 2)	1.514	4.00E-02	experimentally determined, co-expression, protein homology, curated databases, textmining	None
EGR3	early growth response 3	1.502	7.12E-03	experimentally determined, co-expression, textmining	Calcineurin-regulated NFAT-dependent transcription in lymphocytes
VGLL3	vestigial like 3 (Drosophila)	1.489	1.48E-03	co-expression, textmining	None
IL13RA2	interleukin 13 receptor, alpha 2	1.480	1.48E-03	curated databases, co-expression, textmining	Jak-STAT signaling pathway
GADD45B	growth arrest and DNA-damage-inducible, beta	1.454	1.43E-02	co-expression, textmining	None
ADAMTS1	ADAM metallopeptidase with thrombospondin type 1 motif, 1	1.445	3.43E-02	textmining	None

JUN	jun proto-oncogene	1.440	3.59E-03	experimentally determined, co-expression, curated databases, textmining	AP-1 transcription factor network, ATF-2 transcription factor network, Calcineurin-regulated NFAT-dependent transcription in lymphocytes, Validated transcriptional targets of AP1 family members Fra1 and Fra2, Calcium signaling in the CD4+ TCR pathway, Signal transduction through il1r, Direct p53 effectors, Osteoclast differentiation, Leishmaniasis, ErbB signaling pathway
IL1B	interleukin 1, beta	1.439	1.17E-02	experimentally determined, co-expression, curated databases, textmining	Cytokine-cytokine receptor interaction, GPCR signaling, IL23-mediated signaling events, NOD-like receptor signaling pathway, Amoebiasis, Hematopoietic cell lineage, Signal transduction through il1r, Graft-versus-host disease, Osteoclast differentiation, Nfkb activation by nontypeable hemophilus influenzae, Malaria, Leishmaniasis
GRHL1	grainyhead-like 1 (Drosophila)	1.407	4.27E-02	experimentally determined, protein homology, textmining	None
MMP1	matrix metallopeptidase 1 (interstitial collagenase)	1.406	7.25E-03	protein homology, curated databases, textmining	AP-1 transcription factor network, Validated transcriptional targets of AP1 family members Fra1 and Fra2
MAFF	v-maf musculoaponeurotic fibrosarcoma oncogene homolog F (avian)	1.402	1.35E-02	experimentally determined, co-expression, textmining	None
BMP2	bone morphogenetic protein 2	1.368	2.94E-03	curated databases, textmining	Cytokine-cytokine receptor interaction, GPCR signaling
NRG1	neuregulin 1	1.352	4.50E-03	co-expresssion, curated databases, textmining	GPCR signaling, ErbB receptor signaling network, ErbB signaling pathway
EDN1	endothelin 1	1.351	3.17E-02	co-expresssion, curated databases, textmining	AP-1 transcription factor network, GPCR signaling, GPCR ligand binding, Signaling by GPCR, Class A/1 (Rhodopsin-like receptors), Peptide ligand-binding receptors, GPCR downstream signaling
PELI1	pellino E3 ubiquitin protein ligase 1	1.314	5.75E-03	curated databases, textmining	None
ADRB2	adrenoceptor beta 2, surface	1.309	5.81E-03	experimentally determined, curated databases, textmining	GPCR ligand binding, Signaling by GPCR, Class A/1 (Rhodopsin-like receptors), GPCR downstream signaling
RND3	Rho family GTPase 3	1.299	8.48E-03	experimentally determined, co-expression, textmining	None
HAS2	hyaluronan synthase 2	1.296	3.09E-02	textmining	None

ADAMTS6	ADAM metallopeptidase with thrombospondin type 1 motif, 6	1.265	9.78E-03	textmining	None
GRHL3	grainyhead-like 3 (Drosophila)	1.242	2.72E-03	experimentally determined, protein homology, textmining	None
