# Supplementary material for: Group A Streptococcus M1T1 Intracellular Infection of Primary Tonsil Epithelial Cells Dampens Levels of Secreted IL-8 Through the Action of SpyCEP
Source: Front Cell Infect Microbiol. 2018 May 17;8:160. doi: 10.3389/fcimb.2018.00160 (PMC5966554; doi:10.3389/fcimb.2018.00160)
Supplement: Table S3 — Metadata for transcriptome interaction network and pathway analysis of 5448 intracellularly infected TEpi cells in comparison to JRS4 infected cells. Pathway over-representation analysis performed using InnateDB of all differentially expressed genes (adjusted P < 0.05, Log2FC >1 or <-1). All significantly over-represented pathways are shown (adjusted P < 0.05). Protein-protein interaction data type from stringDB output described. [file Table_3.pdf]

Table S3: Metadata for transcriptome interaction network and pathway analysis of 5448 intracellularly infected Tepi cells in comparison to JRS4 infected cells.

Gene symbol	Full name	Log2FC gene expression in comparison to JRS4-infected cells	Adjusted P-value	Protein-protein interaction data type from Stringdb.com	Upregulated pathways from Innatedb.com
FOSB	FBJ murine osteosarcoma viral oncogene homolog B	3.484	3.04E-04	experimentally determined, textmining	Osteoclast differentiation, AP-1 transcription factor network
EGR4	early growth response 4	3.457	1.02E-03	experimentally determined, textmining	Calcineurin-regulated NFAT-dependent transcription in lymphocytes, Downstream signaling in naïve CD8+ T cells
IRF1	interferon regulatory factor 1	3.390	6.49E-04	experimentally determined, coexpression, curated databases, textmining	Glucocorticoid receptor regulatory network, Cytokine Signaling in Immune system, IL6-mediated signaling events, Innate Immune System
TRAF1	TNF receptor-associated factor 1	3.253	6.49E-04	experimentally determined, coexpression, curated databases, textmining	CD40/CD40L signaling, Tnfr2 signaling pathway, HIV-1 Nef: Negative effector of Fas and TNF-alpha, RANKL, TNF receptor signaling pathway, TWEAK
BIRC3	baculoviral IAP repeat containing 3	2.712	6.49E-04	experimentally determined, coexpression, curated databases, textmining	CD40/CD40L signaling, NOD-like receptor signaling pathway, HIV-1 Nef: Negative effector of Fas and TNF-alpha, Toll-Like Receptors Cascades, Activated TLR4 signalling, Toll Like Receptor 4 (TLR4) Cascade, TNF receptor signaling pathway, Innate Immune System

TNF	tumor necrosis factor	2.622	6.54E-04	coexpression, textmining	Calcineurin-regulated NFAT-dependent transcription in lymphocytes, Osteoclast differentiation, Cytokine-cytokine receptor interaction, MAPK signaling pathway, NOD-like receptor signaling pathway, JAK STAT pathway and regulation, HIV-1 Nef: Negative effector of Fas and TNF-alpha, Downstream signaling in naïve CD8+ T cells, T cell receptor signaling pathway, Hematopoietic cell lineage, Nfat and hypertrophy of the heart, Toll-like receptor signaling pathway, IL23-mediated signaling events, TNF receptor signaling pathway, TWEAK, Adipocytokine signaling pathway, GPCR signaling
PTX3	pentraxin 3, long	2.578	3.73E-03	textmining	None
NFKBIE	nuclear factor of kappa light polypeptide gene enhancer in B-cells inhibitor, epsilon	2.468	2.12E-04	experimentally determined, coexpression, textmining	T cell receptor signaling pathway, Adipocytokine signaling pathway
CD83	CD83 molecule	2.433	1.09E-03	coexpression, textmining	None
EGR2	early growth response 2	2.407	8.64E-04	experimentally determined, coexpression, textmining	Calcineurin-regulated NFAT-dependent transcription in lymphocytes, IL4-mediated signaling events
GDF15	growth differentiation factor 15	2.350	2.50E-03	textmining	Direct p53 effectors
CCL20	chemokine (C-C motif) ligand 20	2.308	1.01E-03	curated databases, coexpression, textmining	Cytokine-cytokine receptor interaction, Class A/1 (Rhodopsin-like receptors), GPCR ligand binding, Peptide ligand-binding receptors, Chemokine receptors bind chemokines, Signaling by GPCR, GPCR downstream signaling
LIF	leukemia inhibitory factor	2.261	2.55E-03	curated databases, textmining	Cytokine-cytokine receptor interaction, JAK STAT pathway and regulation, Direct p53 effectors, Nfat and hypertrophy of the heart, GPCR signaling
MAFF	v-maf musculoaponeurotic fibrosarcoma oncogene homolog F (avian)	2.203	6.49E-04	experimentally determined, textmining	None

NR4A3	nuclear receptor subfamily 4, group A, member 3	2.202	4.58E-03	experimentally determined, textmining	None
BCL2A1	BCL2-related protein A1	2.139	8.09E-04	experimentally determined, curated databases, textmining	None
PPP1R15A	protein phosphatase 1, regulatory subunit 15A	2.136	1.26E-04	textmining	None
TNFAIP2	tumor necrosis factor, alpha-induced protein 2	2.114	6.85E-04	textmining	None
NR4A1	nuclear receptor subfamily 4, group A, member 1	2.107	2.78E-03	experimentally determined, coexpression, textmining	MAPK signaling pathway, Glucocorticoid receptor regulatory network, TCR, Innate Immune System
HBEGF	heparin-binding EGF-like growth factor	2.044	6.71E-04	curated databases, textmining	Signaling by GPCR, Nfat and hypertrophy of the heart, GPCR signaling, Innate Immune System
CX3CL1	chemokine (C-X3-C motif) ligand 1	2.007	6.49E-04	textmining	Cytokine-cytokine receptor interaction, Class A/1 (Rhodopsin-like receptors), GPCR ligand binding, Peptide ligand-binding receptors, Chemokine receptors bind chemokines, Direct p53 effectors, Signaling by GPCR
GEM	GTP binding protein overexpressed in skeletal muscle	1.951	8.52E-03	experimentally determined, textmining	None
EGR1	early growth response 1	1.894	3.28E-02	experimentally determined, curated databases, protein homology, textmining	Calcineurin-regulated NFAT-dependent transcription in lymphocytes, AP-1 transcription factor network, Glucocorticoid receptor regulatory network, Downstream signaling in naïve CD8+ T cells, Oncostatin_M, TCR, Cytokine Signaling in Immune system
PTGER4	prostaglandin E receptor 4 (subtype EP4)	1.891	7.20E-04	textmining	Class A/1 (Rhodopsin-like receptors), GPCR ligand binding, Signaling by GPCR, GPCR downstream signaling
ICAM1	intercellular adhesion molecule 1	1.872	1.22E-03	curated databases, coexpression, textmining	Glucocorticoid receptor regulatory network, Toll-Like Receptors Cascades, Activated TLR4 signalling, Toll Like Receptor 4 (TLR4) Cascade, Cytokine Signaling in Immune system, Toll-like receptor signaling pathway, Innate Immune System

BDKRB1	bradykinin receptor B1	1.866	6.73E-03	curated databases	Class A/1 (Rhodopsin-like receptors), GPCR ligand binding, Peptide ligand-binding receptors, Signaling by GPCR, GPCR downstream signaling
GADD45B	growth arrest and DNA-damage-inducible, beta	1.825	1.03E-03	textmining	MAPK signaling pathway, IL12-mediated signaling events
CXCL2	chemokine (C-X-C motif) ligand 2	1.820	3.96E-03	coexpression, curated databases	Cytokine-cytokine receptor interaction, NOD-like receptor signaling pathway, Class A/1 (Rhodopsin-like receptors), GPCR ligand binding, Peptide ligand-binding receptors, Chemokine receptors bind chemokines, Signaling by GPCR, GPCR downstream signaling
EGR3	early growth response 3	1.780	6.68E-04	experimentally determined, textmining	Calcineurin-regulated NFAT-dependent transcription in lymphocytes
TNFAIP3	tumor necrosis factor, alpha-induced protein 3	1.699	1.70E-03	experimentally determined, coexpression, textmining	CD40/CD40L signaling, NOD-like receptor signaling pathway, Tnfr2 signaling pathway, TNF receptor signaling pathway, Innate Immune System
NFKBIZ	nuclear factor of kappa light polypeptide gene enhancer in B-cells inhibitor, zeta	1.647	2.48E-03	experimentally determined, textmining	None
CORO6	coronin 6	1.621	1.22E-03	experimentally determined, coexpression, textmining	None
LRIG3	leucine-rich repeats and immunoglobulin-like domains 3	-1.608	1.02E-03	experimentally determined, textmining	None
ACTL10	actin-like 10	-1.765	2.91E-03	experimentally determined, coexpression, textmining	None
HIST1H2BF	histone cluster 1, H2bf	-1.959	4.94E-02	experimentally determined, coexpression, textmining	None
