# Supplementary figures and images for: Group A Streptococcus M1T1 Intracellular Infection of Primary Tonsil Epithelial Cells Dampens Levels of Secreted IL-8 Through the Action of SpyCEP
Source: Front Cell Infect Microbiol. 2018 May 17;8:160. doi: 10.3389/fcimb.2018.00160 (PMC5966554; doi:10.3389/fcimb.2018.00160)

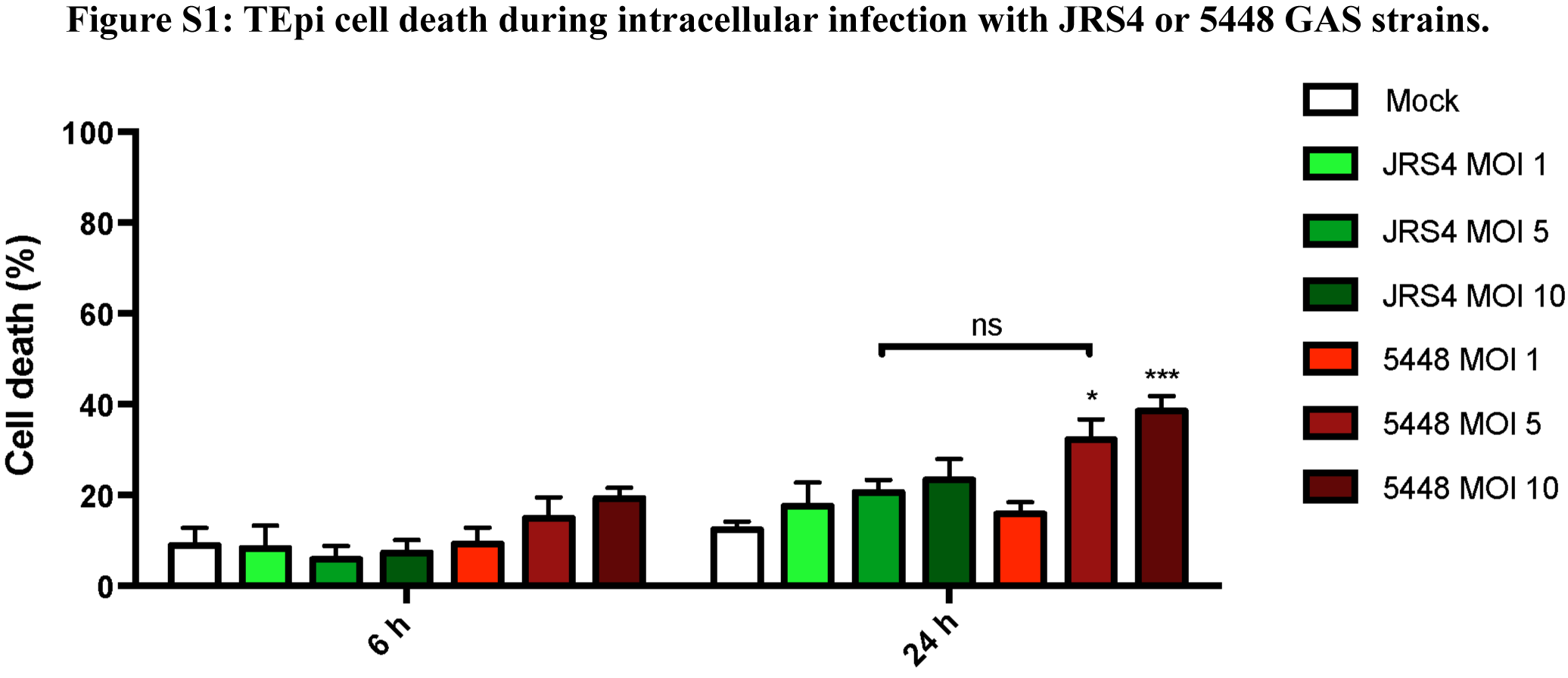

Supplement: Figure S1 — TEpi cell death during intracellular infection with JRS4 or 5448 GAS strains. Cell death measured as percentage of LDH released from TEpi cells after 6 or 24 h following GAS infection. Data are plotted as the mean ± s.e.m. and represent three independent experiments performed in triplicate and analyzed by two-way ANOVA with Tukey's post-test. Significance shown is relative to mock, unless otherwise indicated. *P < 0.05; ***P < 0.001. [file Image_1.tif]

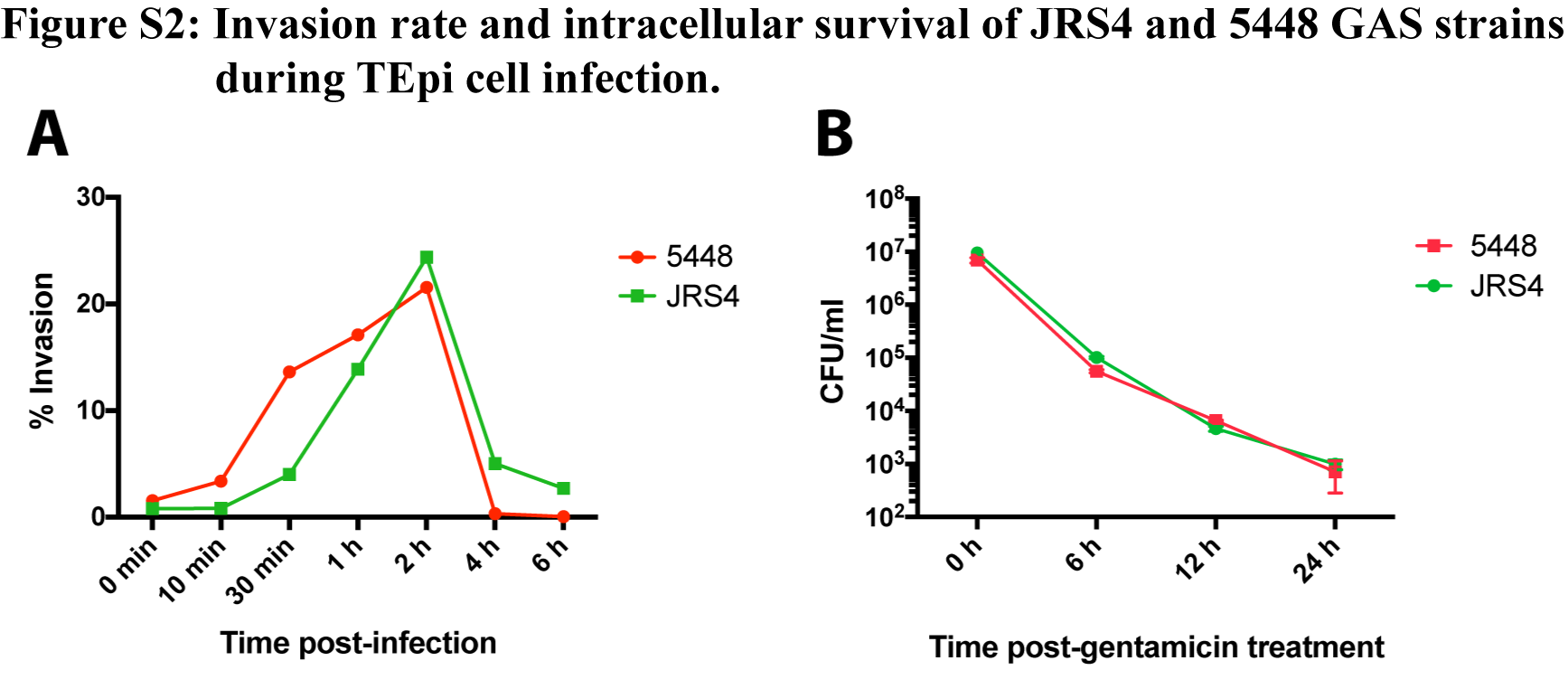

Supplement: Figure S2 — Invasion rate and intracellular survival of JRS4 and 5448 GAS strains during TEpi cell infection. Confluent TEpi cells were infected with either GAS strain at an MOI of 5. (A) Invasion rate was measured at each time post-infection by lysing TEpi cells with 0.2% Triton X-100, before performing a colony forming unit (CFU) assay. TEpi cells infected in parallel were washed and treated with gentamicin for 2 h, before being lysed and CFU assay performed. The invasion rate was measured by dividing the CFU counts of gentamicin treated TEpi cells by non-gentamicin treated wells at each time point. (B) Intracellular survival of GAS was measured by infecting confluent TEpi cells with either GAS strain for 2 h, before replacing the media with gentamicin-containing media for the duration of the experiment. At each time point post-infection, TEpi cells were lysed with 0.2% Triton X-100 and CFU assay performed. Results are representative of three independent experiments. [file Image_2.tif]

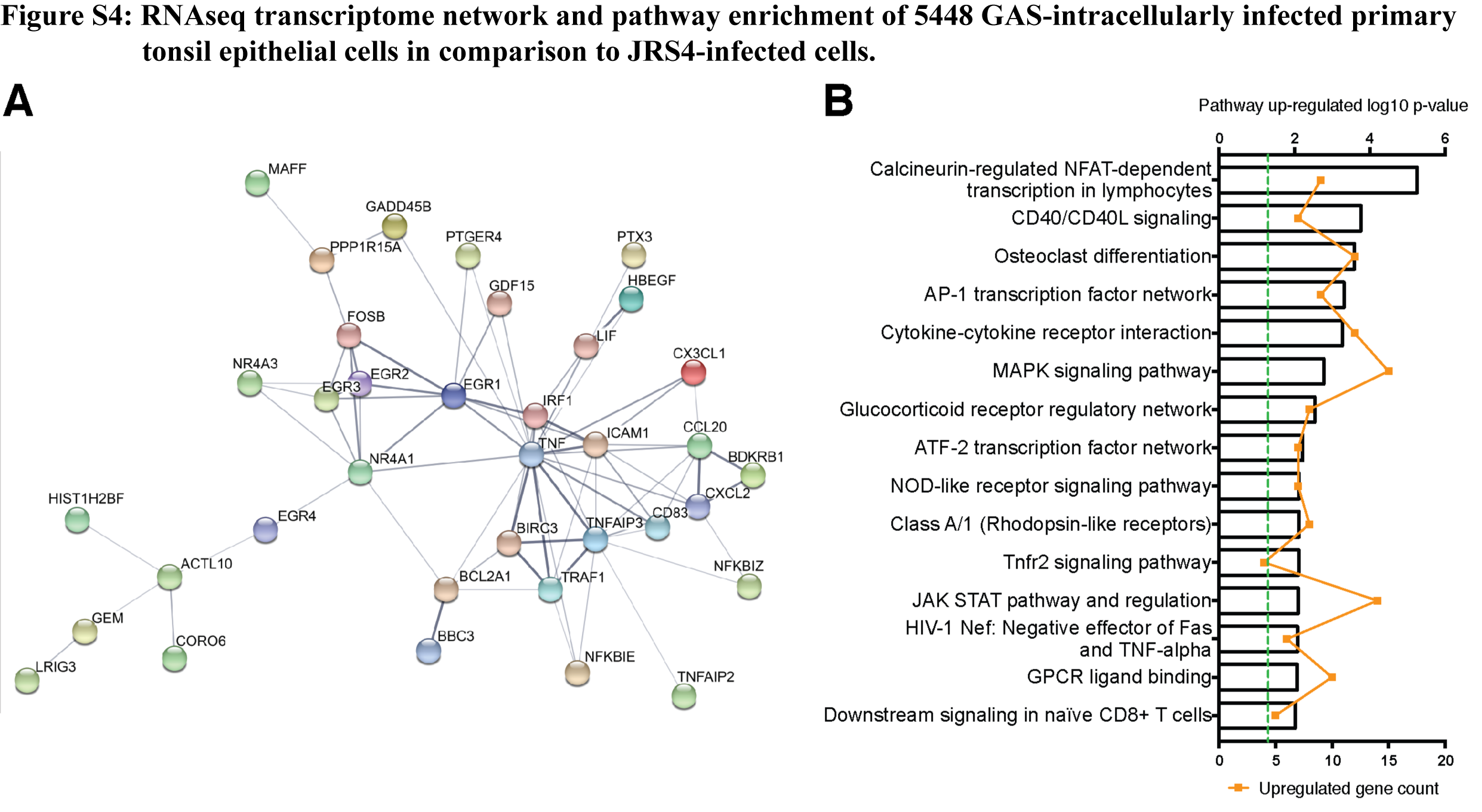

Supplement: Figure S4 — RNAseq transcriptome network and pathway enrichment of 5448 GAS-intracellularly infected primary tonsil epithelial cells in comparison to JRS4-infected cells. (A) Protein-protein interaction network from the top 100 differentially expressed genes (at an adjusted P < 0.05) for 5448-intracellularly infected TEpi cells in comparison to JRS4-infected TEpi cells, generated using STRINGdb (http://string-db.org/). Network edges show the confidence of interactions, where the line thickness indicates the strength of data support. Active interaction sources include textmining, experiments, databases, co-expression, neighborhood, gene fusion and co-occurrence. Minimum required interaction score of medium confidence (0.400). Non-protein coding genes and disconnected nodes are not shown. Node color is arbitrary. (B) Pathway over-representation analysis of all differentially expressed genes (adjusted P < 0.05, Log2FC >1 or <-1) was performed using innatedb.com, for 5448-infected TEpi cells in comparison to JRS4-infected cells. Top 15 up-regulated pathways are shown. Green line indicates threshold for significance. Metadata from analysis results shown in Table S3. [file Image_4.tif]
