# Supplementary material for: Group A Streptococcus M1T1 Intracellular Infection of Primary Tonsil Epithelial Cells Dampens Levels of Secreted IL-8 Through the Action of SpyCEP
Source: Front Cell Infect Microbiol. 2018 May 17;8:160. doi: 10.3389/fcimb.2018.00160 (PMC5966554; doi:10.3389/fcimb.2018.00160)
Supplement: Figure S3 — Amino acid sequence alignment between the cepA genes of 5448 and JRS4. The amino acid residues required for serine protease activity are highlighted (red boxes). An asterisk (*) indicates positions which have a conserved residue, a colon (:) and green lettering indicates conservative amino acid changes, and a period (.) and blue lettering indicates semi-conservative changes. Non-conservative changes are indicated by red lettering. 5448 GenBank accession number: CP008776, SpyCEP protein ID: AKK70939; JRS4 GenBank accession number: CP011414, SpyCEP protein ID: AKI75695. [file Image_3.PDF]

```
             10        20        30        40        50        60
              |         |         |         |         |         |
5448 MEKKQRFSLRKYKSGTFSVLIGSVFLVM-TTTVAADELSTMSEPTITNHAQQQAQHLTNT
JRS4 MEKKQRFSLRKYKSGTFSVLIGSVFLMMMTTTVAADELTTTSEPTITNHAQQQAQHLTNT
     **************************:* *********:* *******************

             70        80        90       100       110       120
              |         |         |         |         |         |
     ELSSAESKSQDTSQITLKTNREKEQSQDLVSEPTTTELADTDAASMANTGSDATQKSASL
     ELSSAESKPQDTSQITPKTNREKEQSQDLVSEPTTTELADTDAASMANTGPDATQKSASL
     ********.******* *********************************.*********

            130       140       150       160       170       180
              |         |         |         |         |         |
     PPVNTDVHDWVKTKGAWDKGYKGQGKVVAVIDTGIDPAHQSMRISDVSTAKVKSKEDMLA
     PPVNTDVHDWVKTKGAWDKGYKGQGKVVAVIDTGIDPAHQSMRISDVSTAKVKSKEDMLA
     ************************************************************

            190       200       210       220       230       240
              |         |         |         |         |         |
     RQKAAGINYGSWINDKVVFAHNYVENSDNIKENQFEDFDEDWENFEFDAEAEPKAIKKHK
     RQKAAGINYGSWINDKVVFAHNYVENSDNIKENQFEDFDEDWENFEFDAEAEPKAIKKHK
     ************************************************************

            250       260       270       280       290       300
              |         |         |         |         |         |
     IYRPQSTQAPKETVIKTEETDGSHDIDWTQTDDDTKYESHGMHVTGIVAGNSKEAAATGE
     IYRPQSTQAPKETVIKTEETDGSHDIDWTQTDDDTKYESHGMHVTGIVAGNSKEAAATGE
     ************************************************************

            310       320       330       340       350       360
              |         |         |         |         |         |
     RFLGIAPEAQVMFMRVFANDIMGSAESLFIKAIEDAVALGADVINLSLGTANGAQLSGSK
     RFLGIAPEAQVMFMRVFANDVMGSAESLFIKAIEDAVALGADVINLSLGTANGAQLSGSK
     ********************:***************************************

            370       380       390       400       410       420
              |         |         |         |         |         |
     PLMEAIEKAKKAGVSVVVAAGNERVYGSDHDDPLATNPDYGLVGSPSTGRTPTSVAAINS
     PLMEAIEKAKKAGVSVVVAAGNERVYGSDHDDPLATNPDYGLVGSPSTGRTPTSVAAINS
     ************************************************************

            430       440       450       460       470       480
              |         |         |         |         |         |
     KWVIQRLMTVKELENRADLNHGKAIYSESVDFKDIKDSLGYDKSHQFAYVKESTDAGYNA
     KWVIQRLMTVKELENRADLNHGKAIYSESVDFKNIKDSLGYDKSHQFAYVKESTDAGYNA
     *********************************:**************************

            490       500       510       520       530       540
              |         |         |         |         |         |
     QDVKGKIALIERDPNKTYDEMIALAKKHGALGVLIFNNKPGQSNRSMRLTANGMGIPSAF
     QDVKGKIALIERDPNKTYDEMIALAKKHGALGVLIFNNKPGQSNRSMRLTSNGMGIPSAF
     **************************************************:*********

            550       560       570       580       590       600
              |         |         |         |         |         |
     ISHEFGKAMSQLNGNGTGSLEFDSVVSKAPSQKGNEMNHFSNWGLTSDGYLKPDITAPGG
     ISHEFGKAMSQLNGNGTGSLEFDSVVSKAPSQKGNEMNHFSNWGLTSDGYLKPDITAPGG
     ************************************************************

            610       620       630       640       650       660
              |         |         |         |         |         |
     DIYSTYNDNHYGSQTGTSMASPQIAGASLLVKQYLEKTQPNLPKEKIADIVKNLLMSNAQ
     DIYSTYNDNHYGSQTGTSMASPQIAGASLLVKQYLEKTQPNLPKEKIADIVKNLLMSNAQ
     ************************************************************
```

```
             670       680       690       700       710       720
               |         |         |         |         |         |
5448  IHVNPETKTTTSPRQQGAGLLNIDGAVTSGLYVTGKDNYGSISLGNITDTMTFDVTVHNL
JRS4  IHVNPETKTTTSPRQQGAGLLNIDGAVTSGLYVTGKDNYGSISLGNVTDTMTFDVTVHNL
      **********************************************:*************

             730       740       750       760       770       780
               |         |         |         |         |         |
      SNKDKTLRYDTELLTDHVDPQKGRFTLTSHSLKTYQGGEVTVPANGKVTVRVTMDVSQFT
      SNKAKTLRYDTELLTDHVDPQKGRFTLTSRSLKTYQGGEVTVPANGKVTVRVTMDVSQFT
      *** *************************:******************************

             790       800       810       820       830       840
               |         |         |         |         |         |
      KELTKQMPNGYYLEGFVRFRDSQDDQLNRVNIPFVGFKGQFENLAVAEESIYRLKSQGKT
      KELTKQMPNGYYLEGFVRFRDSQDDQLNRVNIPFVGFKGQFENLAVAEESIYRLKSQGKT
      ************************************************************

             850       860       870       880       890       900
               |         |         |         |         |         |
      GFYFDESGPKDDIYVGKHFTGLVTLGSETNVSTKTISDNGLHTLGTFKNADGKFILEKNA
      GFYFDESGPKDDIYVGKHFTGLVTLGSETNVSTKTISDNGLHTLGTFKNADGKFILEKNA
      ************************************************************

             910       920       930       940       950       960
               |         |         |         |         |         |
      QGNPVLAISPNGDNNQDFAAFKGVFLRKYQGLKASVYHASDKEHKNPLWVSPESFKGDKN
      QGNPVLAISPNGDNNQDFAAFKGVFLRKYQGLKASVYHASDKEHKNPLWVSPESFKGDKN
      ************************************************************

             970       980       990      1000      1010      1020
               |         |         |         |         |         |
      FNSDIRFAKSTTLLGTAFSGKSLTGAELPDGHYHYVVSYYPDVVGAKRQEMTFDMILDRQ
      FNSDIRFAKSTTLLGTAFSGKSLTGAELPDGYYHYVVSYYPDVVGAKRQEMTFDMILDRQ
      *******************************:****************************

            1030      1040      1050      1060      1070      1080
               |         |         |         |         |         |
      KPVLSQATFDPETNRFKPEPLKDRGLAGVRKDSAFYLERKDNKPYTVTINDSYKYVSVED
      KPVLSQATFDPETNRFKPEPLKDRGLAGVRKDSVFYLERKDNKPYTVTINDSYKYVSVED
      *********************************.**************************

            1090      1100      1110      1120      1130      1140
               |         |         |         |         |         |
      NKTFVERQADGSFILPLDKAKLGDFYYMVEDFAGNVAIAKLGDHLPQTLGKTPIKLKLTD
      NKTFVERQADGSFILPLDKAKLGDFYYMVEDFAGNVAIAKLGDHLPQTLGKTPIKLKLTD
      ************************************************************

            1150      1160      1170      1180      1190      1200
               |         |         |         |         |         |
      GNYQTKETLKDNLEMTQSDTGLVTNQAQLAVVHRNQPQSQLTKMNQDFFISPNEDGNKDF
      GNYQTKETLKDNLEMTQSDTGLVTNQAQLAVVHRNQPQSQLTKMNQDFFISPNEDGNKDF
      ************************************************************

            1210      1220      1230      1240      1250      1260
               |         |         |         |         |         |
      VAFKGLKNNVYNDLTVNVYAKDDHQKQTPIWSSQAGASVSAIESTAWYGITARGSKVMPG
      VAFKGLKNNVYNDLTVNVYAKDDHQKQTPIWSSQAGASASAIESTAWYGITARGSKVMPG
      **************************************.*********************

            1270      1280      1290      1300      1310      1320
               |         |         |         |         |         |
      DYQYVVTYRDEHGKEHQKQYTISVNDKKPMITQGRFDTINGVDHFTPDKTKALGSSGIVR
      DYQYVVTYRDEHGKEHQKQYTISVNDKKPMITQGRFDTINGVDHFTPDKTKALGSSGIVR
      ************************************************************
```

```
            1330      1340      1350      1360      1370      1380
               |         |         |         |         |         |
5448 EEVFYLAKKNGRKFDVTEGKDGITVSDNKVYIPKNPDGSYTISKRDGVTLSDYYYLVEDR
JRS4 EEVFYLAKKNGRKFDVTEGKDGITVSDNKVYIPKNPDGSYTISKRDGVTLSDYYYLVEDR
     ************************************************************

            1390      1400      1410      1420      1430      1440
               |         |         |         |         |         |
     AGNVSFATLRDLKAVGKDKAVVNFGLDLPVPEDKQIVNFTYLVRDADGKPIENLEYYNNS
     AGNVSFATLRDLKAVGKDKAVVNFGLDLPVPEDKQIVNFTYLVRDADGKPIENLEYYNNS
     ************************************************************

            1450      1460      1470      1480      1490      1500
               |         |         |         |         |         |
     GNSLILPYGKYTVELLTYDTNAAKLESDKIVSFTLSADNNFQQVTFKITMLATSQITAHF
     GNSLILPYGKYTVELLTYDTNAAKLESDKIVSFTLSADNNFQQITFKMTMLATSQITAHF
     *******************************************:***:************

            1510      1520      1530      1540      1550      1560
               |         |         |         |         |         |
     DHLLPEGSRVSLKTAQDQLIPLEQSLYVPKAYGKTVQEGTYEVVVSLPKGYRIEGNTKVN
     DHLLPEGSRVSLKTAQGQLIPLEQSLYVPKAYGKTVQEGTYEVVVSLPKGYRIEGNTKVN
     ****************.*******************************************

            1570      1580      1590      1600      1610      1620
               |         |         |         |         |         |
     TLPNEVHELSLRLVKVGDASDSTGDHKVMSKNNSQALTASATPTKSTTSATAKALPSTGE
     TLPNEVHELSLRLVKVGDASDSTGDHKVMSKNNSQALTASATPTKTTTSATAKALPSAGE
     *********************************************:***********:**

            1630      1640
               |         |
     KMGLKLRIVGLVLLGLTCVFSRKKSTKD
     KMGLKLRIVGLVLLGLTCVFSRKKSTKD
     ****************************
```
